# Identification of RNA-binding proteins that partner with Lin28a to regulate Dnmt3a expression

**DOI:** 10.1038/s41598-021-81429-8

**Published:** 2021-01-27

**Authors:** Silvia Parisi, Daniela Castaldo, Silvia Piscitelli, Chiara D’Ambrosio, Giuseppina Divisato, Fabiana Passaro, Rosario Avolio, Alessia Castellucci, Paolo Gianfico, Mariorosario Masullo, Andrea Scaloni, Tommaso Russo

**Affiliations:** 1 Department of Molecular Medicine and Medical Biotechnology, University of Naples Federico II, 80131 Naples, Italy; 2 Proteomics and Mass Spectrometry Laboratory, ISPAAM, Italian National Research Council, 80147 Naples, Italy; 3 Department of Movement Sciences and Wellbeing, University of Naples Parthenope, 80133 Naples, Italy

**Keywords:** Biochemistry, Cell biology, Molecular biology, Stem cells

## Abstract

Lin28 is an evolutionary conserved RNA-binding protein that plays important roles during embryonic development and tumorigenesis. It regulates gene expression through two different post-transcriptional mechanisms. The first one is based on the regulation of miRNA biogenesis, in particular that of the let-7 family, whose expression is suppressed by Lin28. Thus, loss of Lin28 leads to the upregulation of mRNAs that are targets of let-7 species. The second mechanism is based on the direct interaction of Lin28 with a large number of mRNAs, which results in the regulation of their translation. This second mechanism remains poorly understood. To address this issue, we purified high molecular weight complexes containing Lin28a in mouse embryonic stem cells (ESCs). Numerous proteins, co-purified with Lin28a, were identified by proteomic procedures and tested for their possible role in Lin28a-dependent regulation of the mRNA encoding DNA methyltransferase 3a (*Dnmt3a*). The results show that Lin28a activity is dependent on many proteins, including three helicases and four RNA-binding proteins. The suppression of four of these proteins, namely Ddx3x, Hnrnph1, Hnrnpu or Syncrip, interferes with the binding of Lin28a to the *Dnmt3a* mRNA, thus suggesting that they are part of an oligomeric ribonucleoprotein complex that is necessary for Lin28a activity.

## Introduction

Lin28 was identified initially in *Caenorhabditis elegans* where it regulates the timing of embryo development^1^. Two paralogs have been found in mammals, known as Lin28a and Lin28b, which are expressed in ESCs and embryonic tissues and become undetectable in most adult cells^2,3^. Both proteins play fundamental roles in stem cells^4^ and during the early stages of development^5^; their ectopic expression is able to induce malignant transformation and Lin28a or Lin28b overexpression is frequently associated with cancers^6–9^. The key role of Lin28 in pluripotent cells is also supported by the observations that, together with Oct4, Sox2 and Klf4, Lin28 is able to reprogram adult cells^10^ and that endogenous Lin28a and Lin28b genes are both necessary to achieve high reprogramming efficiency^11^.

Lin28 regulates gene expression by acting as an inhibitor of let-7 miRNA biogenesis, thus promoting the translation of mRNAs that are targeted by members of the let-7 family^12–14^. Mechanistically, Lin28 directly interacts with pre-let-7 and recruits the terminal uridylyltransferases Tut4 or Tut7^15–17^. The latter catalyze the oligouridylylation of pre-let-7 that favors pre-let-7 degradation by the exonuclease Dis3l2^18,19^. In addition to the regulation of let-7 biogenesis, Lin28 also acts through the direct binding to mRNAs that results in the regulation of their translation^20,21^. Lin28 binding can both enhance^22–24^ and suppress translation of mRNAs^25^. Importantly, the let-7-independent function of Lin28 appears to be of great physiological relevance, as exemplified by the regulation of hemoglobin switching^26^.

Although several papers have reported long lists of mRNAs targeted by Lin28^11,24,27–29^, the mechanisms through which Lin28 influences their translation are not definitively understood^30^.

The events that take place in the mouse blastocyst at implantation can be mimicked in vitro by inducing the differentiation of ESCs into epiblast like stem cells (EpiLCs). This differentiation is driven by a complex mechanism, involving the transcription factors Oct4 and Otx2^31,32^. Hmga2 is another of the factors necessary for this differentiation^33^. We have demonstrated that Lin28a and b are induced and tightly regulated during the transition from ESCs into EpiLCs, and that they regulate the translation of *Hmga2* mRNA, in a let-7-independent manner^34^. Considering that Lin28 expression declines when ESCs go beyond the EpiLC state to differentiate into neuroecoderm^34^, these cells appear to be an ideal experimental system in which to study the molecular mechanisms underlying Lin28 functions.

Hundreds of RNA-binding proteins (RBPs) have been identified and many of them are expressed in ESCs^35^, but their mechanisms are still poorly understood. Given that many RBPs bind to the same mRNA molecule^36^, it can be hypothesized that each of them represents a subunit of large molecular machineries that modulate mRNA translation. Through this lens, Lin28 could be considered one of these subunits. To explore this hypothesis, we have purified Lin28a-containing ribonucleoprotein complexes, in native conditions, and identified by proteomics numerous proteins candidate partners of Lin28a. Through an RNA interference-based screen, we have found that some of these candidate partners are necessary for the Lin28-dependent regulation of the *Dnmt3a* mRNA, here identified as a direct target of Lin28a. These results support the hypothesis that Lin28a is part of an oligomeric machinery that regulates mRNA translation.

## Results

### Identification of Lin28 candidate partners in EpiSCs

**Figure 1:**
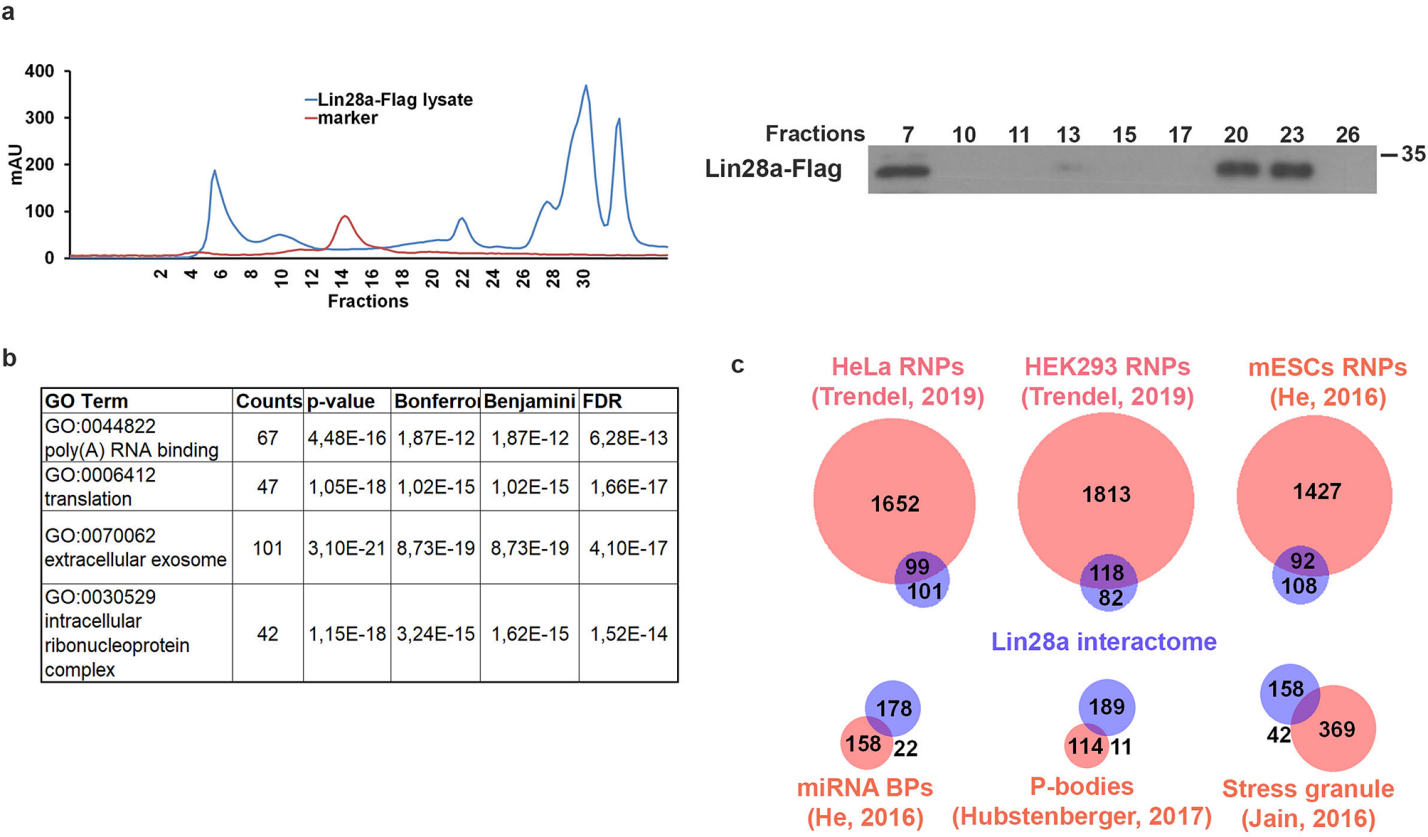
Identification of Lin28a interactors during EpiLC formation. **(a)** Chromatogram (left) showing the elution profile of the size-exclusion chromatography. ESCs were transfected with Lin28a-Flag expressing construct at 1d of EpiLCs transition and the protein extracts obtained at 3d EpiLCs were loaded on a Superose 6 Increase 10/300 GL column. Western blot (right) analysis of column fractions with the anti-Flag antibody shows the presence of ectopic Lin28a protein in specific fractions. **(b)** GO terms (biological process and molecular function) identified using DAVID software for the 201 proteins specifically found in Lin28 immunoprecipitates by proteomic analysis. **(c)** Venn diagram comparing Lin28 co-purified proteins with lists of proteins interacting directly or indirectly with RNA molecules. HeLa RNPs, HEK293 RNPs and ESC RNPs: proteins found in ribonucleoprotein particle of HeLa, HEK293 and ESC cell lines respectively; *miRNA BPs* miRNA binding proteins, *P-bodies* protein found in P-bodies of HEK293 and HeLa cells, *stress granule* proteins found in mammalian cells.

In order to identify candidate partners of Lin28 in mouse EpiSCs, we purified the proteins co-precipitated with Lin28a-Flag. The first step to isolate the Lin28a-containing complexes was based on the purification of native complexes by size-exclusion chromatography. Western blot analysis of the fractions of the Superose 6 Increase 10/300 GL column showed that Lin28 was present in the fractions 6, 7, 8, 20, 21, 22 and 23 (Fig. 1a). The fractions where Lin28 was present were immunoprecipitated with an anti-Flag antibody. Three pools of immunoprecipitates were generated: fractions 6, 7 and 8 (pool 1), 20 and 21 (pool 2) and 22 and 23 (pool 3). Pool 1 was divided into two aliquots that were analyzed separately (pool 1a and pool 1b). The proteins present in the four samples were analyzed by proteomic procedures based on high resolution mass spectrometry. The same procedure was carried out on a protein extract from EpiSCs transfected with an empty vector (mock transfected) and the results were used to identify the background proteins.

We first excluded all the bona fide background proteins, clearly identified in the immunoprecipitates from mock transfected cells. After this filtering, Pools 1, 2 and 3 were demonstrated containing 167, 60 and 50 proteins, respectively (Supplementary Tables 1, 2, and 3). Some of these proteins are present in more than one pool, thus overall, we identified 201 proteins (Supplementary Tables 4 and 5). Lin28-copurified proteins showed that the most significant GO terms were “Poly(A) RNA binding” with 77 out of 201 belonging to this category, and “Translation”, including 47 out of 201 proteins (Fig. 1b).

A more detailed analysis was made by examining the overlap between our list of Lin28 co-purified proteins and those of published lists of proteins interacting directly or indirectly with RNA molecules. Forty-nine proteins of our list are also present in the repertoire of RNA binding proteins detected in mouse ESCs^37^ and most of them are also present in the list of ribonucleoprotein particles (RNPs) of two human cell lines^38^ (Fig. 1c and Supplementary Table 6). Considering the role of Lin28 in the regulation of miRNAs^16^, we also examined the list of the proteins interacting with pre-miRNAs^39^. Twenty-four out of 180 miRNA-binding proteins in various human cell lines were also present in the list of Lin28 co-purified proteins. The latter included 7 Hnrnps also found in the lists of mRNA-interacting proteins (Supplementary Table 6).

Another proteome we examined is that of P-bodies, considering that a relevant fraction of repressed mRNA transits these intracellular structures. Among the proteins most significantly enriched in P-bodies of HEK293 and HeLa cells, only 8 were found in our list, in agreement with the observation that Lin28 is not enriched in P-bodies^40^. The comparison with stress granule proteome showed that 42 of 411 bona fide stress-granule enriched proteins were co-purified with Lin28^41^ (Fig. 1c and Supplementary Table 6).

Proteomic analysis did not identify Tut4 and/or Tut7 in our samples. To confirm the absence of high-molecular weight complexes co-immunoprecipitated with Lin28a containing these two proteins, western blot analyses were performed on Lin28a immunoprecipitates by using Tut4 or Tut7 antibodies. In both cases, no evident signals were present (see Supplementary Fig. 1), thus indicating that Lin28a/Tut4-7 complexes are in a molecular size range excluded from the analyzed fractions.

In conclusion, ontology analysis indicated that about 30% of the proteins co-purified with Lin28 are involved in RNP complexes. This result suggests that the experimental approach we used led to the purification of bona-fide RNA–protein complexes containing Lin28a. To explore this possibility, we carried out the same purification steps described above by using cell extracts pretreated with RNaseA, before to load them onto the Sepharose column. The results of proteomic analysis of the RNaseA experiments showed that 92 proteins present in the pool 1 were lost upon RNaseA pretreatment (Supplementary Table 1). In addition, 31 of these 92 proteins were found in pool 2 or in the pool 3 of the experiment with the RNaseA treatment (Supplementary Table 1). These results indicate that the RNaseA treatment significantly altered the panel of proteins co-purified with Lin28, and in some cases provoked a shift towards smaller fractions, due to the decrease of the molecular size of the complexes. This suggests that some proteins co-immunoprecipitated with Lin28 and lost or shifted to lower size fractions could be considered as candidate partners of Lin28a in RNP complexes.

### Functional screening of proteins co-immunoprecipitated with Lin28a

The relatively large number of proteins identified by the proteomics posed the problem of how to identify those having a functional relationship with Lin28. We reasoned that if one of the proteins co-immunoprecipitated with Lin28a has a role in the function of Lin28a, we can predict that the silencing of this protein should affect the function of Lin28a. The availability of a large collection of shRNAs targeting mouse genes rendered feasible a first rough screening. For the readout of this screening, we developed an assay based on the effect of Lin28 overexpression on the translation of a luciferase reporter construct, where the luc coding sequence is fused to the 3′ UTR of a Lin28 target.

**Figure 2:**
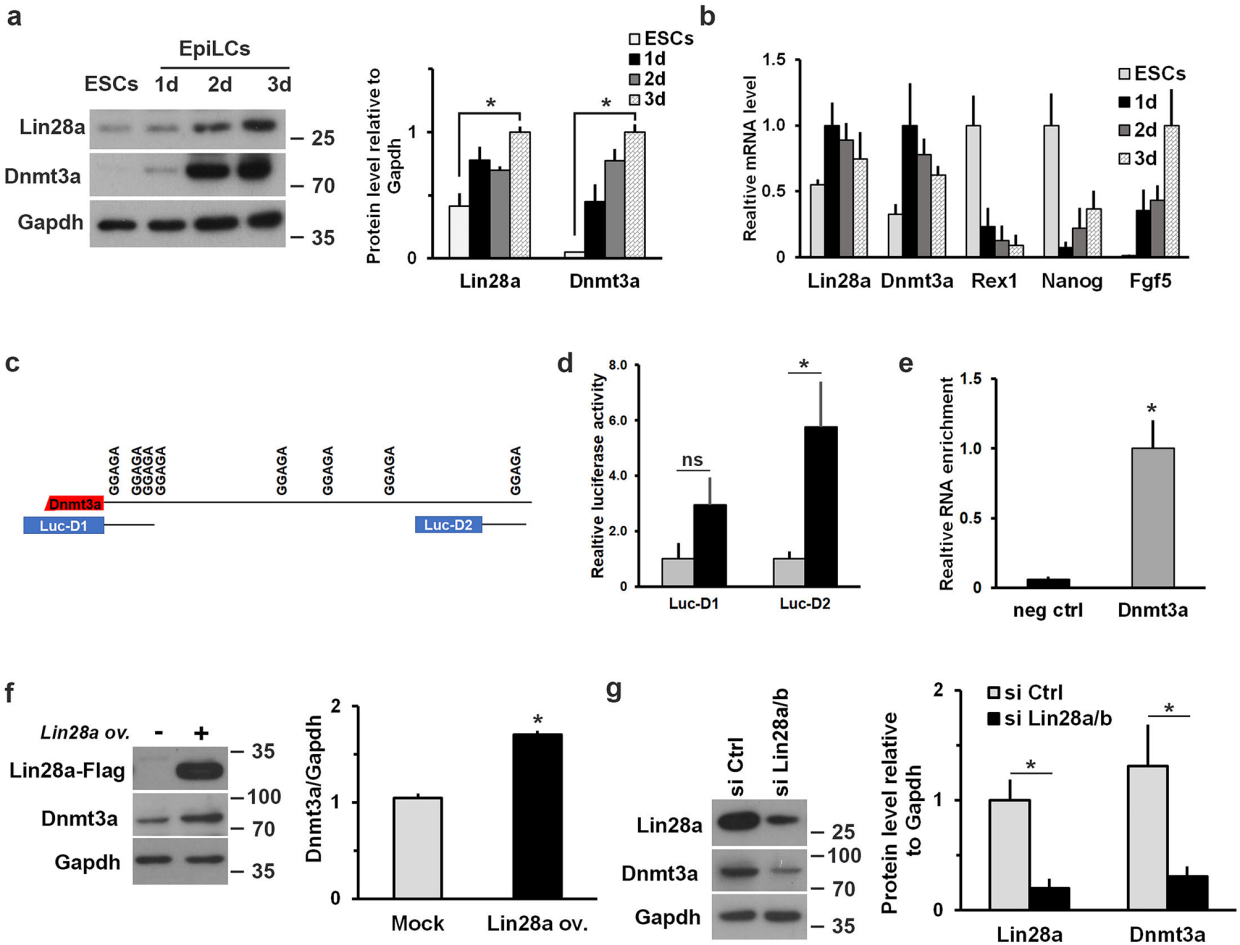
Lin28a regulates Dnmt3a level through direct binding to its mRNA. **(a)** Expression profile of Dnmt3a and Lin28a was analyzed by western blot at different time points during the transition from ESCs to EpiLCs. Graph represents relative band intensity expressed as Dnmt3a or Lin28a intensity relative to Gapdh (n = 3 biological replicates). *P < 0.05 (Student’s t-test, two tailed). **(b)** q-PCR analysis to define the expression profile of *Dnmt3a* mRNA during the establishment of EpiLCs. Expression of ESC (*Rex1* and *Nanog*) and EpiLC (*Nanog* and *Fgf5*) markers is reported. The data are presented as fold changes relative to the highest value reached ± SEM of three independent experiments. **(c)** Schematic representation of the two regions cloned downstream the luciferase reporter and containing possible Lin28 binding sites. **(d)** Results of the luciferase assay showing the effects of Lin28a overexpression using the two luciferase constructs. After 1 day of EpiLC transition, the cells were co-transfected with Lin28a-Flag expressing plasmid or empty vector (Mock), and Luc-D1 or Luc-D2 constructs. Data on luciferase activity, normalized to Renilla luciferase, are shown as mean ± SEM of fold changes relative to values measured in Mock transfected cells. P value of biological replicates (n ≥ 4) was calculated using Student’s t-test (two tailed). *P < 0.05; ns: not significant. **(e)** Cells at 2 day of EpiLC transition were transfected with Lin28a-Flag expressing plasmid or the empty vector and were collected at 3 days (EpiLCs) to perform RNA immunoprecipitation analysis. Binding of Lin28a protein to *Dnmt3a* mRNA was measured by qPCR on reverse-transcribed immunoprecipitated RNAs. Pri-miR-23a, expressed in both ESCs and EpiLCs, was used as negative control. Data are represented as mean fold change ± SEM of the immunoprecipitated RNA in Lin28 overexpressing sample (n = 4 biological replicates). *P < 0.05 (Student’s t-test, two tailed). **(f)** Western blot analysis of Dnmt3a protein in 3 days EpiLCs upon transfection of Lin28a-Flag or empty vector at 1 day of transition. Western blot analysis with anti-Flag antibody demonstrates the overexpression of Lin28a-Flag protein. Graph represents relative band intensity expressed as Dnmt3a intensity relative to Gapdh (n = 3 biological replicates). *P < 0.05 (Student’s t-test, two tailed). **(g)** Western blot analysis of Dnmt3a protein level in 3d EpiLCs transfected with non-targeting (si Ctrl) or Lin28a/b targeting siRNAs at 1 day of transition. Western blot analysis with Lin28a antibody shows Lin28 silencing. Graph represents relative band intensity expressed as Dnmt3a or Lin28a intensity relative to Gapdh (n = 3 biological replicates). *P < 0.05 (Student’s t-test, two tailed).

As mentioned above, there are many papers reporting the systematic analysis of mRNAs that are targets of Lin28. Among them the DNA methyltransferase 3a (*Dnmt3a*) emerges as a prime candidate. Indeed, the expression profile of Dnmt3a protein phenocopies that of Lin28a, with a strong accumulation during transition from undifferentiated mouse ESCs into EpiLCs^42^ (Fig. 2a), which is not completely paralleled by a similar change in *Dnmt3a* mRNA levels (Fig. 2b). At neural precursor stage (SFEBs) Lin28a is still expressed whereas Dnmt3a levels decrease. Then, both proteins disappear when the cells undergo neuronal differentiation (Supplementary Fig. 2a). Moreover, the KO of *Dnmt3a* in ESCs showed the same delay in the exit from the naïve state observed in Lin28 KO cells^43^, due to its function in regulating this process^11, 43^.

The 3′ UTR of *Dnmt3a* mRNA contains several putative sequences that could interact with Lin28. We have generated two luciferase reporter constructs bearing two regions of *Dnmt3a* mRNA containing possible Lin28 binding sites (Luc-D1 and Luc-D2, Fig. 2c). To exclude a let-7 dependent effect, we analyzed these regions for the presence of possible let-7 binding by using TARGETSCAN and RNA22 programs; this analysis confirmed that no putative let-7 sites are present in these two regions. As shown in Fig. 2d, the co-expression of Lin28a-Flag and the Luc-D2 construct induced a significant increase of luciferase activity compared to the control.

This effect of Lin28a overexpression was almost completely abolished when we used a deletion mutant of Luc-D2 construct (Supplementary Fig. 2b) thus indicating the sequence-dependent specificity of Lin28a effect on *Dnmt3a* 3′UTR. The demonstration of the direct interaction of Lin28a with the *Dnmt3a* mRNA came from RNA immunoprecipitation experiments. As shown in Fig. 2e, endogenous *Dnmt3a* mRNA co-immunoprecipitated with Lin28. The functional effect of Lin28a modulation of endogenous Dnmt3a expression was demonstrated by the accumulation of Dnmt3a protein upon Lin28a overexpression in EpiLCs (Fig. 2f). Accordingly, the silencing of Lin28a led to an evident decrease of Dnmt3a accumulation at the protein level (Fig. 2g). Moreover, the level of Dnmt3a protein can be rescued upon Lin28a silencing by re-expressing Lin28a (Supplementary Fig. 2c).

**Figure 3:**
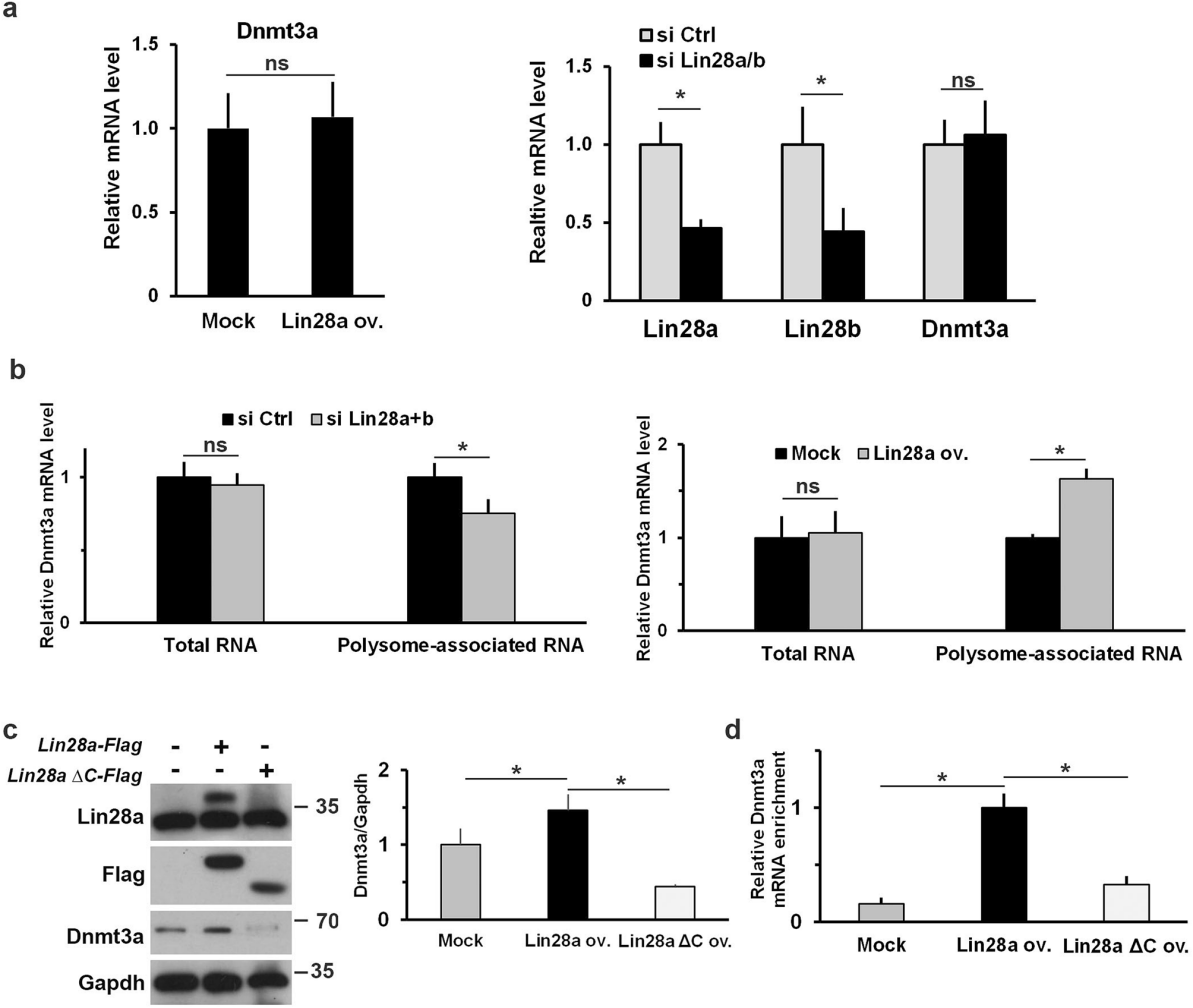
Lin28a acts on *Dnmt3a* translation. **(a)** qPCR analysis to measure levels of *Dnmt3a* mRNA upon Lin28a overexpression or Lin28a/b silencing in EpiLCs. The levels of *Lin28* a and *Lin28b* were measured as silencing efficiency control. Data are shown as means ± SEM of fold changes relative to values measured in control cells (n ≥ 3). *P < 0.05; ns: not significant (Student’s t-test, two tailed). **(b)** ESCs induced to EpiLC transition were transfected at 2 days with plasmids or siRNAs as indicated. The level of *Dnmt3a* mRNA was measured by qPCR in EpiLCs both in input samples (total RNA) and the polysome-associated fractions. Data are shown as means ± SEM of fold changes relative to values measured in control cells (n ≥ 3). *P < 0.05; ns: not significant (Student’s t-test, two tailed). **(c)** Wild type and C-terminal deleted (ΔC) forms of Lin28a were overexpressed in EpiLCs and the level of Dnmt3a was measured by western blot analysis. Graph represents relative band intensity expressed as Dnmt3a intensity relative to Gapdh. (n = 3 biological replicates). *P < 0.05 (Student’s t-test, two tailed). The protein signal detected with anti-Flag antibody identifies bands of the ectopic Lin28a-Flag and Lin28a ΔC-Flag proteins. **(d)** Cells at 2 day of EpiLC transition were transfected with Lin28a-Flag or Lin28a ΔC-Flag expressing plasmid or the empty vector and were collected at 3 days (EpiLCs) to perform RNA immunoprecipitation analysis. Binding of Lin28a and Lin28a ΔC proteins to *Dnmt3a* mRNA was measured by qPCR on reverse-transcribed immunoprecipitated RNAs. Data are represented as mean fold change ± SEM of the immunoprecipitated RNA in Lin28a overexpressing sample (n = 4 biological replicates). *P < 0.05 (Student’s t-test, two tailed).

The effect of Lin28a modulation on Dnmt3a protein level appeared to be independent from transcriptional regulation, because Lin28a overexpression or KD did not change *Dnmt3a* mRNA levels (Fig. 3a). To further demonstrate that Lin28a controls translation of *Dnmt3a*, we have analyzed the translation status of *Dnmt3a* mRNA upon silencing or overexpression of Lin28a in EpiLCs through polysome profiling. Polysome profiling is a well-established assay to evaluate translational efficiency, where increased association of mRNA with polysomes indicates increased translation efficiency, and vice versa. The analysis of *Dnmt3a* mRNA found in the isolated polysome fractions showed a significant increase upon Lin28a overexpression and a decrease upon Lin28a silencing (Fig. 3b). Of note, the polysome gradient profile is not changed (Supplementary Fig. 3) neither upon Lin28a overexpression nor its silencing thus indicating that Lin28a controls the degree of *Dnmt3a* mRNA associated with active translation in a substrate-specific manner and not through a more general effect on translation.

A dominant negative mutant has been studied by Ju et colleagues in human HEK293 cells^30^. This mutant, lacking the C-terminal region of human Lin28a, exerts a dominant-negative effect on Lin28-dependent stimulation of translation. We have generated a similar mutant of mouse Lin28a protein (Lin28a ΔC) and we have analyzed the effect on binding activity to *Dnmt3a* mRNA and on Dnmt3a protein abundance. Despite the efficient expression of Lin28a ΔC protein in EpiLCs, the overexpression of this mutant did not result in accumulation of Dnmt3a protein but instead led to a sensible decrease of endogenous Dnmt3a (Fig. 3c). RIP analysis showed that the deletion mutant is not able to efficiently bind *Dnmt3a* mRNA (Fig. 3d). These results indicated that the C-terminal domain is required for Lin28a to bind *Dnmt3a* mRNA and thus to control *Dnmt3a* translation and confirm previous observation^30^ that Lin28a ΔC can function as dominant negative on Lin28-dependent stimulation of translation.

**Figure 4:**
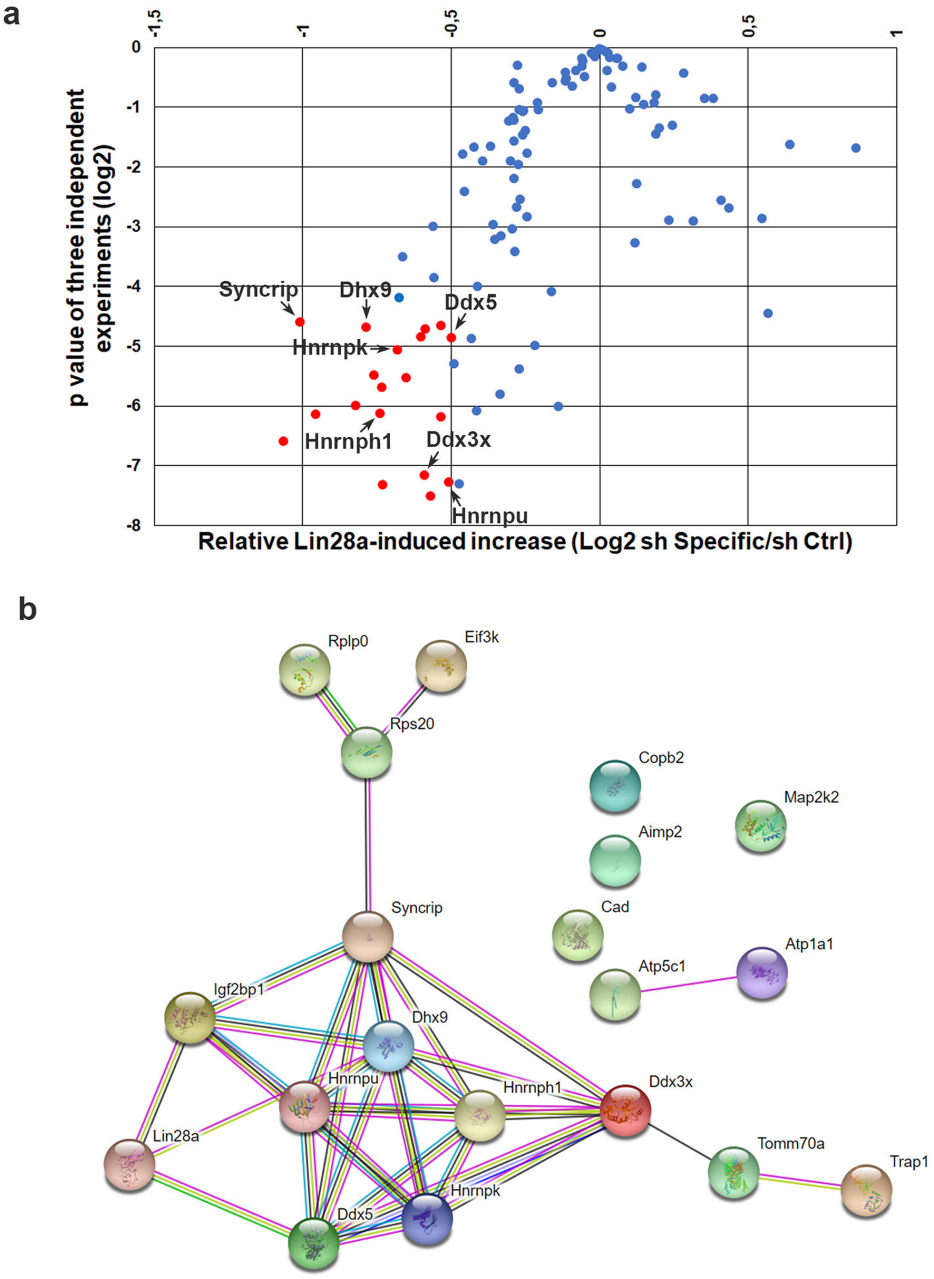
RNA interference screening to identify functional partners of Lin28a. **(a)** Volcano plot representing the results of the luciferase screening to identify Lin28a functional partners. Cells at 1 day of EpiLC transition were co-transfected with Lin28a-Flag expressing plasmid, with Luc-D2 and with control or specific shRNAs. Luciferase activity was measured in EpiLCs (3 days). Data on luciferase activity, normalized to Renilla luciferase, are shown as mean ± SEM of fold changes relative to values measured in sh Ctrl transfected cells. P value of biological replicates (n = 3) was calculated using Student’s t-test (two tailed). Red bullets represent data with *P < 0.05 and showing a decrease of at least 30% of luciferase accumulation due to the silencing of interactors compared to cells transfected with the non-silencing shRNA. **(b)** Result of STRING analysis showing the protein–protein association network of Lin28a and selected functional partners.

Based on all these data we have used Dnmt3a as tool to find possible functional partners of Lin28a. To this aim, we analyzed the levels of the Luc-D2 construct in EpiLCs transfected with Lin28a and with single shRNA, each targeting one of 109 selected candidate partners (see Supplementary Table 7). These candidate partners were selected on the basis of the availability of at least one cognate shRNA in the library. The analysis of luciferase levels showed that the silencing of numerous candidate partners impairs the effect of Lin28a overexpression on the accumulation of luciferase (Fig. 4a, and Supplementary Table 8). Indeed, in 19 out of 109 silenced genes, the effect of Lin28a overexpression was decreased by at least 30% compared to cells transfected with the non-silencing shRNA, and the differences were significant with a P ≤ 0.05 (Fig. 4a, red bullets). These 19 proteins, together with Lin28a, were examined by using the STRING platform that allowed us to identify a network of 8 proteins, that are: Ddx3x, Ddx5, Dhx9, Igf2bp2, Hnrnph1, Hnrnpk, Hnrnpu and Syncrip (Fig. 4b).

### Helicases and RNA binding proteins play a role in Lin28a-dependent regulation of Dnmt3a expression

**Figure 5:**
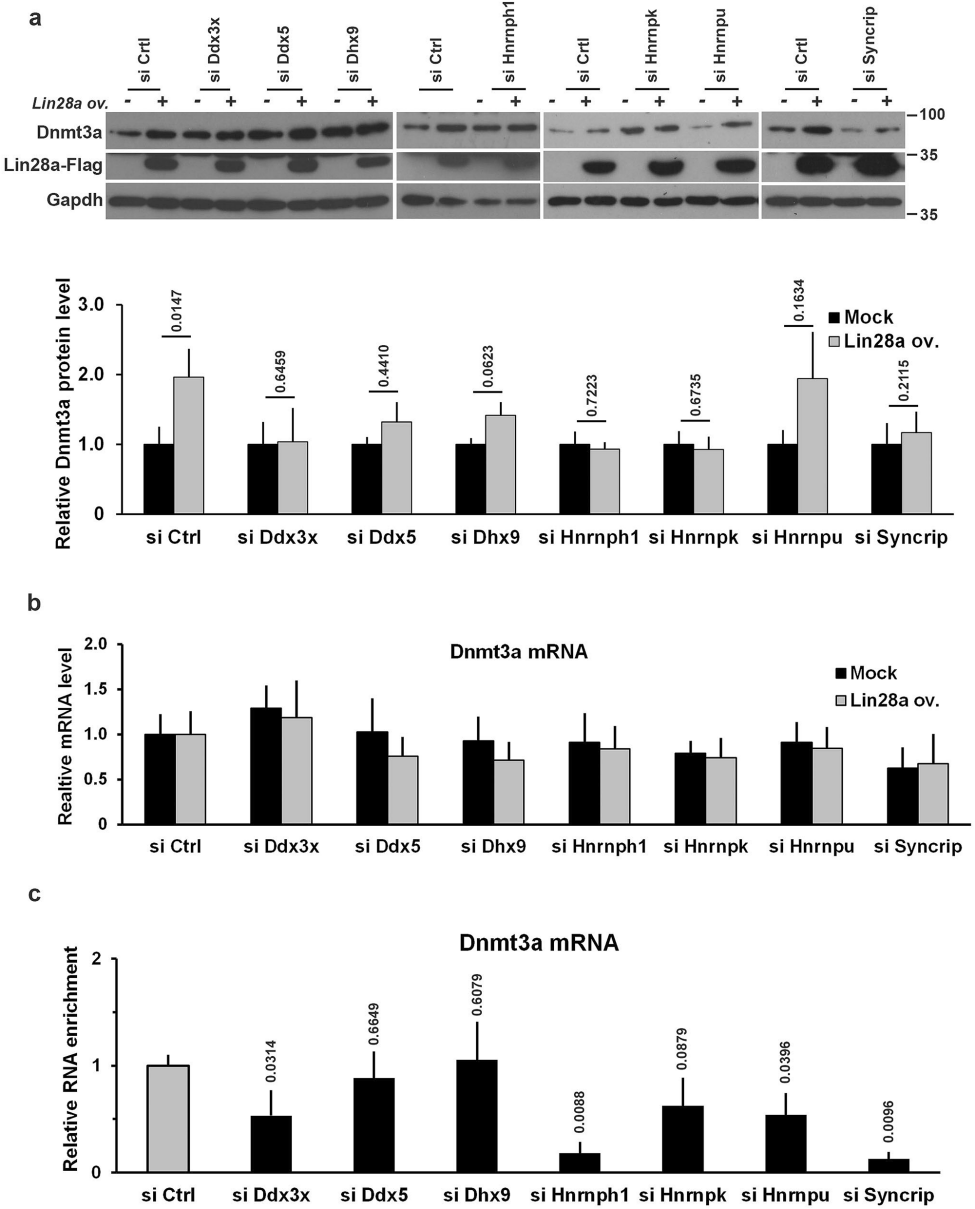
Helicases and RNA binding proteins influence Lin28a-dependent regulation of Dnmt3a expression. **(a)** Western blot analysis showing the effects of silencing of Lin28a interactors on the accumulation of Dnmt3a protein upon Lin28a overexpression in EpiLCs. Cells at 1 day of EpiLC transition were co-transfected with Lin28a-Flag expressing plasmid or empty vector (mock) and non-targeting or specific siRNAs, and were collected at 3 days (EpiLCs). Graph represents relative band intensity expressed as Dnmt3a intensity relative to Gapdh (n ≥ 3 biological replicates). The data are expressed as fold changes of the values measured in the relative control cells. P value (Student’s t-test, two tailed) is reported on the relative bar. **(b)** qPCR analysis to measure levels of *Dnmt3a* mRNA upon silencing of Lin28a interactors (si Specific) in presence of Lin28a overexpression in EpiLCs. Cells at 1 day of EpiLC transition were co-transfected with plasmid expressing Lin28a-Flag or empty vector and non-targeting or specific siRNAs, and were collected at 3 days (EpiLCs). Data are expressed as means ± SEM of fold changes compared to the *Dnmt3a* value in Mock si Control transfected cells (n ≥ 3). Student’s t-test (two tailed) performed comparing the values of *Dnmt3a* mRNA among the Mock and Lin28 ov. samples upon silencing of specific interactors showed not statistically significant differences. No significant differences were also found by comparing the *Dnmt3a* mRNA of si Specific versus si Control in both Mock and Lin28 ov. samples. **(c)** Cells at 2 day of EpiLC transition were co-transfected with plasmid expressing Lin28a-Flag and control or specific siRNAs and were collected at 3 days (EpiLCs) to perform RNA immunoprecipitation analysis. Binding of Lin28a to *Dnmt3a* mRNA was measured by qPCR on reverse-transcribed immunoprecipitated RNAs. Data are represented as mean ± SEM of the fold change of the immunoprecipitated RNA relative si Ctrl transfected cells (n = 3 biological replicates). P value (Student’s t-test, two tailed) is reported on the relative bar.

The results described above suggested the occurrence of proteins co-immunoprecipitated with Lin28a whose silencing affects the Lin28a-mediated regulation of translation. To explore the possible in vivo role of these proteins, we decided to examine the effects of their suppression on the translation of endogenous *Dnmt3a* mRNA. To this aim, we selected the three helicases (Ddx3x, Ddx5 and Dhx9) and the four RNA binding proteins (Hnrnph1, Hnrnpk, Hnrnpu and Syncrip) and we first assessed their expression during the transition from ESCs to EpiLCs (Supplementary Fig. 4). We then suppressed the expression of these Lin28a partners by transfecting the cells at 1 day of EpiLC transition with specific siRNAs and analyzed the effects of silencing (see Supplementary Fig. 5a) on the levels of endogenous Dnmt3a protein in EpiLCs. The silencing of any of these proteins had an evident effect on the accumulation of Dnmt3a protein as a consequence of Lin28a overexpression (Fig. 5a and Supplementary Fig. 5b). This phenomenon did not depend on decreased levels of the *Dnmt3a* mRNA, which was not affected by the silencing of the proteins under examination (Fig. 5b).

These observations suggested a close cross talk between Lin28a and each of these proteins. One potential mechanism based on our finding is that the binding of Lin28a to the *Dnmt3a* mRNA is dependent on the availability of these proteins, likely through their association with the target mRNA. To address this possibility, we examined the co-immunoprecipitation of *Dnmt3a* mRNA with Lin28a in cells where Lin28a partners had been suppressed. As shown in Fig. 5c, the silencing of either Ddx3x, Hnrnph1, Hnrnpu or of Syncrip decreased or even eliminated the RIP of *Dnmt3a* mRNA with Lin28a-Flag. The silencing of these partners also significantly impairs the ability of Lin28a overexpression to increase the luciferase activity of Luc-D2 (Supplementary Fig. 5c). Considering that the amount *Dnmt3a* mRNA was not affected by the silencing of these proteins (Figs. 5c), we concluded that they are required for, or at least favor, the binding of Lin28a to the *Dnmt3a* mRNA.

## Discussion

Numerous RNA-binding proteins have been demonstrated to affect the translation of specific arrays of mRNAs, mostly through sequence-specific mechanisms. One example of RNA-binding proteins regulating the translation of specific mRNAs is that of Lin28 proteins. Indeed, in addition their well-characterized role in repression of let-7 miRNA biogenesis, they also bind directly to a large array of mRNAs and regulate their translation. The mechanisms of action of Lin28 bound to specific mRNAs are still not completely understood. Here, we have demonstrated that Lin28a interacts with the mRNA encoding the DNA methyltransferase Dnmt3a and regulates its translation in EpiLCs. Considering that Lin28a overexpression resulted in the accumulation of endogenous Dnmt3a and, accordingly, Lin28 suppression caused a downregulation of Dnmt3a, in both cases without affecting mRNA levels, the Lin28a-dependent *Dnmt3a* mRNA regulation appears to be an ideal system with which to explore Lin28a regulation of translation. Towards this end, we have purified Lin28a containing high-molecular weight complexes in native conditions. This allowed us to identify numerous proteins, including some already known RNA-binding proteins, which were co-immunoprecipitated with Lin28a. Given that the 3′UTR of *Dnmt3a* mRNA contains at least two regions where Lin28a associates and that luciferase constructs containing these regions are translated with different efficiencies in cells overexpressing Lin28a, we screened the proteins co-immunoprecipitated with Lin28a for their contribution to Lin28a-dependent regulation of Luc constructs containing the *Dnmt3a* 3′UTR. This screening demonstrated that the silencing of numerous proteins coimmunoprecipitated with Lin28a affects the Lin28a-dependent regulation of luciferase constructs.

We focused our attention on several proteins already known to interact with mRNAs, comprising three RNA helicases and four RNA binding proteins. By analyzing the effects of their silencing on endogenous *Dnmt3a* mRNA, we observed that the silencing of any of these seven proteins prevented the accumulation of Dnmt3a resulting from Lin28a overexpression. These results suggest functional linkage between Lin28a and each of these factors. RNA immunoprecipitation experiments showed that four Lin28a partners, Ddx3x, Hnrnph1, Hnrnpu and Syncrip, appear to be necessary for Lin28a interaction with *Dnmt3a* mRNA. They could act through various mechanisms. Ddx3x is an ATP-dependent DEAD-box RNA helicase, having various roles in translation^44–46^, which could favor the interaction of Lin28 with its mRNA targets by unwinding mRNA secondary structures that limit Lin28a interaction with its mRNA targets. In hESCs, inhibition of Ddx3x also downregulates critical pluripotency markers (Oct4, Sox2 and Nanog) and facilitates differentiation^47^. However, a general consensus on the possible role of this helicase activity in vivo, if any, is still lacking^48^. Another possibility is that Ddx3x, Hnrnph1, Hnrnpu and Syncrip could be building blocks of RNA-based platforms where Lin28a is recruited. Hnrnph1 and Hnrnpu and Syncrip (also known as Hnrnpq) are RNA-binding proteins. Syncrip regulates mRNA translation^49–51^, splicing^52,53^, localization^54^ and turnover rate^55^. Less is known about the role of Hnrnph1 and Hnrnpu, as most of the studies on these proteins focused on their role in the nucleus. However, their binding to mRNAs was already reported^56,57^.

The co-immunoprecipitation of Ddx3x, Hnrnph1, and Syncrip with Lin28a is RNA-dependent (Supplementary Table 1). Therefore, the hypothesis of the oligomeric complex including them and Lin28a cannot be excluded, although it is still to be demonstrated.

Ddx5, Dhx9 and Hnrnpk silencing did not affect the co-immunoprecipitation of Lin28a with *Dnmt3a* mRNA. This result excludes the possibility that these three proteins are necessary for the binding of Lin28a to the mRNA. Nevertheless, the observation that their silencing has a robust effect on Lin28a-dependent regulation of *Dnmt3a* mRNA strongly supports the hypothesis that mRNA regulation is dependent on large macromolecular complexes and not only on the effects of isolated single proteins^58^. In conclusion, we speculate that these proteins may function as cofactors of Lin28a to specify its RNA substrates and to modulate its effect in fine-tuning of mRNA translation.

## Methods

### Plasmid generation

For gene reporter assay, two different regions of *Dnmt3a* 3′UTR (D1 and D2: nucleotides 1–825 and 5008–5730 of *Dnmt3a* 3′UTR), bearing possible Lin28 binding sites, were cloned downstream firefly luciferase reporter gene (Luc-D1 and Luc-D2) in pCAG-Luc vector. The two regions were obtained by PCR using ESC genomic DNA as template and the oligonucleotides carrying KpnI restriction site as primers (see sequences in Supplementary Table 9).

Luc-D2 deletion mutants are generated by PCR using Luc-D2 as template and the oligonucleotides carrying KpnI restriction site as primers (see sequences in Supplementary Table 9). Both were cloned downstream firefly luciferase reporter gene in pCAG-Luc vector.

For the generation of Lin28a ΔC mutant the pCAG Lin28a-Flag plasmid was used as template^34^. The Lin28a ΔC sequence coding the amminoacids 1–182 was obtained by PCR using the oligonucleotides reported in Supplementary Table 9 and was cloned upstream FLAG-tag in the p-CAG-FLAG vector by using BamHI and HindIII restriction sites.

### Cell culture, generation of EpiLCs and transfection

E14Tg2a (BayGenomics) mouse ESCs were grown on gelatine-coated plates in the following ESC medium: Glasgow minimum essential medium (Sigma-Aldrich) supplemented with 2 mM glutamine, 1 mM sodium pyruvate, 1 × nonessential amino acids (all from Thermo Fisher Scientific), 0.1 mM β-mercaptoethanol (Sigma-Aldrich), 10% fetal bovine serum (Hyclone Laboratories), and 10^3^ U/ml leukaemia inhibitory factor (EMD Millipore).

EpiLCs were obtained from ESCs as previously described^34^. Briefly, ESCs were dissociated into a single-cell suspension, and 40.000 cells/cm^2^ were plated on Geltrex (Thermo Fisher Scientific) -coated plates in the following EpiLC medium: 1:1 vol of DMEM/F-12 and Neurobasal medium, supplemented with 0.5% N2 and 1% B27 supplements, 1% KO serum replacement, 2 mM glutamine (all from Thermo Fisher Scientific), 20 ng/ml activin A, and 12 ng/ml basic fibroblast growth factor (PreproTech).

The differentiation into neurons through serum free embryoid body (SFEB) formation was obtained as previously described^34^.

Transfections of small interfering RNAs (shRNAs, siRNAs) (Open BioSystems and Thermo Fisher Scientific) and plasmids were performed using Lipofectamine 2000 (Thermo Fisher Scientific) following the manufacturer’s instructions. All experiments were performed by transfecting the cells at 1 day of EpiLC transition and the samples were collected and analysed at 3 days when the EpiLCs were established.

### RNA isolation and quantitative PCR

Total RNA was extracted by Tri-Sure (Bioline) and first-strand cDNA was synthesized using Mu-MLV RT (New England Biolabs) according to the manufacturer’s instructions. qPCR was carried out with the QuantStudio 7 Flex using Fast SYBR Green PCR Master Mix (Thermo Fisher Scientific)^34^. The housekeeping *Gapdh* mRNA was used as an internal standard for normalization. Gene-specific primers used for amplification are listed in Supplementary Table 9. qPCR data are presented as fold changes relative to the indicated reference sample using 2^DeltaCt comparative analysis^34^.

### RNA immunoprecipitation and co-immunoprecipitation

Mouse EpiLCs were transfected with pCAG-Lin28a-Flag or Lin28a ΔC-Flag or empty vector and when indicated with specific siRNAs at 2 days of EpiLC induction. EpiLCs (3 days) were harvested and resuspended in polysome lysis buffer with protease and RNAse inhibitors (Sigma-Aldrich) and then centrifuged to remove cell debris. One milligram of total proteins was immunoprecipitated with anti-Flag affinity gel (Sigma-Aldrich) in the following NT2 buffer: 50 mM Tris–HCl pH 7.5,150 mM NaCl, 1 mM MgCl_2_, 0.05% NP-40. After washing the beads were treated with proteinase K (10 mg/ml; Sigma-Aldrich) for 1 h at 55 °C to allow the release of bound RNA. To recover bound RNA the beads were treated with TRIsure, and first-strand cDNA synthesis and qPCR were carried out as indicated above. The qPCR results were analyzed relating the Ct of each sample to the Ct of the input sample (DCt = Ct (input) – Ct (IP)) and then applying 2^DeltaCt comparative analysis. Oligonucleotide pairs used are listed in Supplementary Table 9.

To analyze Lin28a interacting proteins we used the same protocol of RNA immunoprecipitation described above to safeguard RNA-dependent complexes. To recover protein complexes, after washing, the anti-Flag affinity gel beads were resuspended in 2 × Laemmli buffer and boiled for 10 min. Then the beads were discarded, and the supernatant was loaded on SDS-PAGE for western blotting.

### Western blot

Whole cell extracts were isolated using 1× passive lysis buffer (Promega) and protease inhibitors (Sigma-Aldrich). Total proteins were quantified using Bradford Protein Assay (Bradford Reagent, Bio-Rad). For western blot analysis, the proteins were separated by SDS-PAGE under reducing conditions, transferred on PVDF membranes (Merk Millipore) and incubated with the following primary antibodies: anti-Flag (Sigma-Aldrich), anti-Dnmt3a (Cell Signalling Technology and Santa Cruz), anti-Lin28a (Abcam and Cell Signalling Technology), anti-Gapdh, Ddx3x, Ddx5, Ddh9, Hnrnph1, Hnrnpk, Hnrnpu (all from Santa Cruz Biotechnology), anti-Tut-4, anti-Tut-7 and anti-Syncrip (all from Proteintech).

### Size exclusion chromatography

To isolate the Lin28a-containing complexes the cells were transfected with Lin28a-Flag or empty vector at 1 day of EpiLC transition and collected in polysome lysis buffer to safeguard both protein–protein and RNA–protein interactions. To isolate RNA independent complexes the cell lysates were treated with 40 µg/ml of RNAse A for 30′ at room temperature. To remove cell debris all the lysates were centrifuged at high speed (18.000 RPM) using Beckman TLA 120.2.

Then, cell lysates were run on Superose 6 Increase 10/300 GL column at 5 MPa and 0.5 ml/min flow rate in the following buffer: KCl 100 mM, TrisCl pH 7.8 20 mM, MgCl_2_ 5 mM. Fractions of 0.5 ml were collected and concentrated through precipitation with cold acetone to be loaded on SDS-PAGE for western blot analysis or immunoprecipitated with anti-Flag affinity gel for proteomic analysis.

### Proteomic analysis

Immunopurified proteins were analyzed by 12% T SDS-PAGE. After staining with colloidal Coomassie blue, whole gel lanes were cut into 12 slices, minced and washed with water. Corresponding proteins were separately *in-gel* reduced, *S*-alkylated with iodoacetamide and digested with trypsin, as previously reported^59^. Individual protein digests were then analyzed with a nanoLC-ESI-Q-Orbitrap-MS/MS platform consisting of an UltiMate 3000 HPLC RSLC nano system (Thermo Fisher Scientific) coupled to a Q-ExactivePlus mass spectrometer through a Nanoflex ion source (Thermo Fisher Scientific)^59^. Peptides were loaded on an Acclaim PepMap RSLC C18 column (150 mm × 75 μm ID, 2 μm particles, 100 Å pore size) (Thermo Fisher Scientific), and eluted with a gradient of solvent B (19.92/80/0.08 v/v/v water/acetonitrile/formic acid) in solvent A (99.9/0.1 v/v water/formic acid), at a flow rate of 300 nl/min^59^. The gradient of solvent B started at 3%, increased to 40% over 40 min, raised to 80% over 5 min, remained at 80% for 4 min, and finally returned to 3% in 1 min, with a column equilibrating step of 30 min before the subsequent chromatographic run. The mass spectrometer operated in data-dependent mode using a full scan (*m/z* range 375–1,500, a nominal resolution of 70,000, an automatic gain control target of 3,000,000, and a maximum ion target of 50 ms), followed by MS/MS scans of the 10 most abundant ions. MS/MS spectra were acquired in a scan *m/z* range 200–2000, using a normalized collision energy of 32%, an automatic gain control target of 100,000, a maximum ion target of 100 ms, and a resolution of 17,500^59^. A dynamic exclusion value of 30 s was also used. Duplicate analysis of each sample was performed to increase the number of identified peptides/protein coverage.

MS and MS/MS raw data files per lane were merged for protein identification into Proteome Discoverer v. 2.1 software (Thermo Fisher Scientific), enabling the database search by Mascot algorithm v. 2.4.2 (Matrix Science) with the following parameters: UniProtKB mouse protein database (84,433 sequences) including the most common protein contaminants; carbamidomethylation of Cys as fixed modification; oxidation of Met, deamidation of Asn and Gln, and pyroglutamate formation of Gln as variable modifications. Peptide mass tolerance and fragment mass tolerance were set to ± 10 ppm and ± 0.05 Da, respectively. Proteolytic enzyme and maximum number of missed cleavages were set to trypsin and 2, respectively. Protein candidates assigned on the basis of at least two sequenced peptides and Mascot score ≥ 30 was considered confidently identified. Definitive peptide assignment was always associated with manual spectra visualization and verification^59^. Results were filtered to 1% false discovery rate. A comparison with results from the corresponding control allowed to identify contaminant proteins in each experiment that, nonetheless their abundance, were removed from the list of Lin28-interacting partners (Supplementary Tables 1, 2 and 3).

Extended proteomic data are available upon request.

### Luciferase reporter assays

For luciferase assay, Lin28-Flag or empty vector was co-transfected with Luciferase constructs and shRNAs or siRNA as indicated. After 48 h from transfection (3d EpiLCs), the cells were lysed, and firefly and Renilla luciferase activities (as an internal control) were measured with the dual luciferase reporter system (Promega) by Sirius Luminometer (Berthold Detection Systems). The data were expressed as relative to the indicated controls after normalization to Renilla luciferase reading.

### Polysome profiling

For polysome profiling 2 × 10 cm plates of EpiLCs transfected as indicated in the Figure were incubated for 15 min at 37 °C with fresh EpiLC medium supplemented with 100 µg*/*ml of cycloheximide (Sigma-Aldrich). Cells were then washed with ice cold phosphate-buffered saline (PBS) supplemented with 100 µg/ml cycloheximide (Sigma-Aldrich) and resuspended in 300 µl of lysis buffer (10 mM Tris–HCl pH 7.4, 100 mM KCl, 10 mM MgCl2, 1% Triton-X100 (AppliChem), 40 U/ml Recombinant Ribonuclease Inhibitor (Invitrogen), 1 mM DTT (Sigma-Aldrich), Protease Inhibitor Cocktail (Sigma-Aldrich), 100 µg/ml of cycloheximide. After 30 min of incubation on ice, cell lysate was centrifuged for 10 min at 13.000 rpm at 4° C. The supernatant was collected, and samples were normalized for both total RNA and total protein with UV5 Nano (Mettler Toledo). Equal amounts of cell lysate were loaded onto a 10–50% sucrose gradient obtained by adding 6 ml of 10% sucrose over a layer of 6 ml 50% sucrose prepared in lysis buffer without Triton, in a 12-ml tube (Polyallomer; Beckman Coulter)^60^. Gradients were obtained with a gradient maker (Gradient Master, Biocomp Instruments). Polysomes were separated by centrifugation at 37.000 rpm for 2 h using a Beckmann SW41Ti rotor. Twelve fractions of 930 µl were collected with Piston Gradient Fractionator Biocomp (Model 152/153). Polysomes were identified by reading the absorbance at 254 nm. Polysome-containing fractions (6–12) were treated with Tri-Sure (Bioline) to extract the total RNA and pooled. RNA isolation, reverse transcription and quantitative PCR were performed as indicated above.

### Statistical analysis

The number of biological replicates of each experiment is indicated in the figure legends. The means of at least three independent experiments were used to calculate SEM and to perform statistical analysis. All P values were calculated by Student’s t test using a two-tailed test and paired samples^34^.

## Supplementary Information

Supplementary Information.

## Data Availability

The datasets generated during and/or analysed during the current study are available from the corresponding author on reasonable request.
