# Supplementary material for: Identification of RNA-binding proteins that partner with Lin28a to regulate Dnmt3a expression
Source: Sci Rep. 2021 Jan 27;11:2345. doi: 10.1038/s41598-021-81429-8 (PMC7841167; doi:10.1038/s41598-021-81429-8)
Supplement: Supplementary file 1 — Supplementary Information. [file 41598_2021_81429_MOESM1_ESM.pdf]

Identification of RNA-binding proteins that partner with Lin28a to regulate Dnmt3a expression

Silvia Parisi[1]*, Daniela Castaldo[1], Silvia Piscitelli[1], Chiara D'Ambrosio[2], Giuseppina Divisato[1], Fabiana Passaro[1], Rosario Avolio[1], Alessia Castellucci[1], Paolo Gianfico[1], Mariorosario Masullo[3], Andrea Scaloni[2], and Tommaso Russo[1]*

[1] Department of Molecular Medicine and Medical Biotechnology, University of Naples Federico II, 80131 Naples, Italy
[2] Proteomics & Mass Spectrometry Laboratory, ISPAAM, Italian National Research Council, 80147 Naples, Italy
[3] Department of Movement Sciences and Wellbeing, University of Naples Parthenope, 80133 Naples, Italy

*Corresponding authors: silvia.parisi@unina.it, tommaso.russo@unina.it

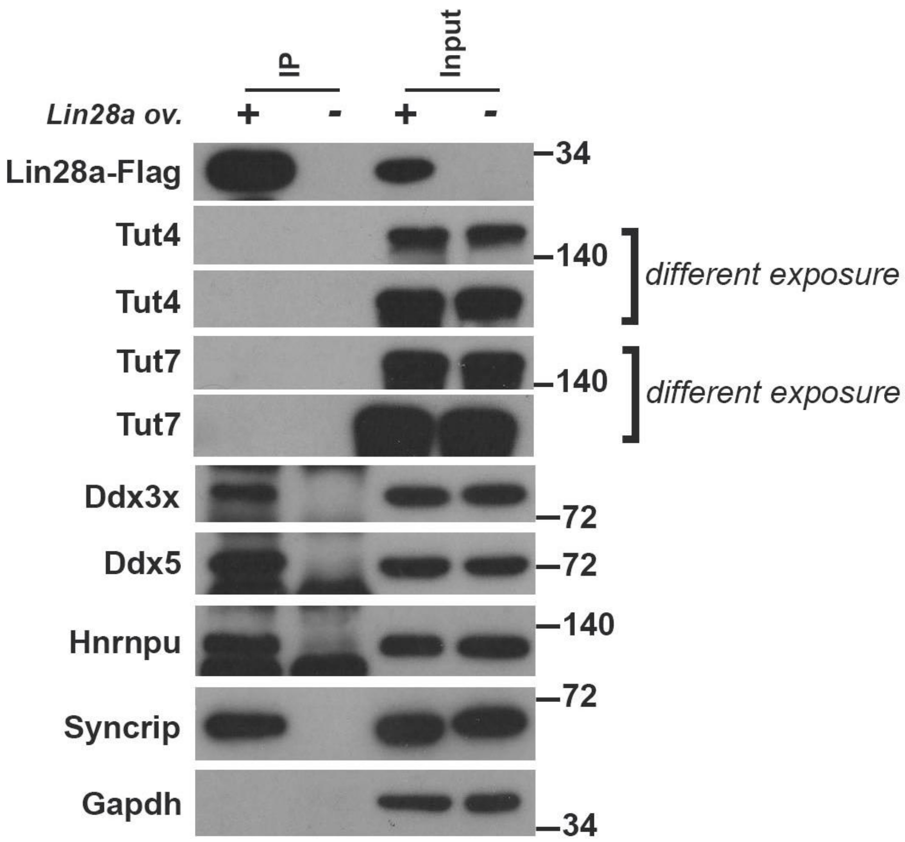

Supplementary Figure 1: Tut4 and Tut7 proteins did not immunoprecipitate with Lin28a in EpiLCs.

Western blot analysis of Lin28a immunoprecipitation showing that Tut4 and Tut7 do not interact with Lin28a during EpiLC transition. Cells at 2 day of EpiLC transition were transfected with Lin28a-Flag expressing vector or empty plasmid, and the immunoprecipitation for the Flag-tag was performed in established EpiLCs (3 days). The binding of Ddx3x, Ddx5, Hnrnpu and Syncrip was analyzed as positive control (see further on in the manuscript). Gapdh is the negative control.

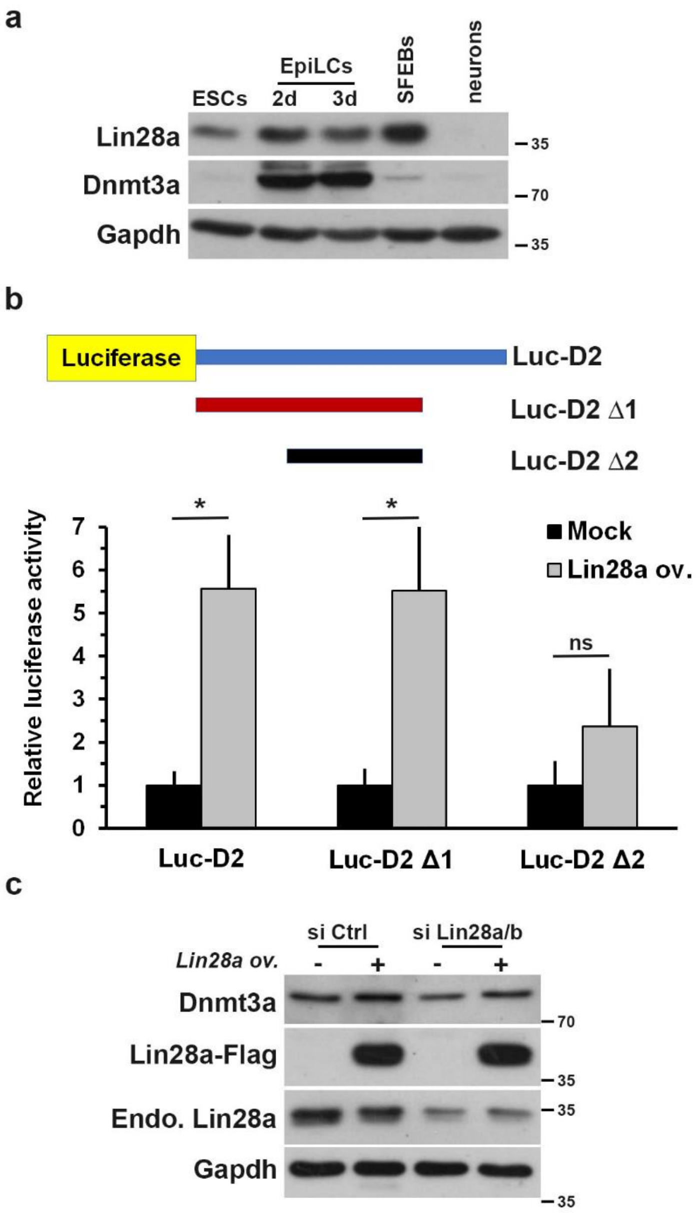

Supplementary Figure 2: Lin28a controls Dnmt3a.

(a) Expression profile of Lin28a and Dnmt3a during ESC differentiation. SFEBs (serum free embryoid bodies) represent neural precursor stage, neurons correspond to 10 days differentiated cells (through SFEB formation) when post-mitotic neurons develop. (b) Schematic representation of the two deletion mutants (top panel) derived from Luc-D2 construct. Graph represents the luciferase activity in EpiLCs upon overexpression of Lin28a. After 1 day of EpiLC transition, the cells were co-transfected with Lin28a-Flag expressing plasmid or empty vector (Mock), and Luc-D2 or deletion mutants. The Luc-D2 Δ1 form excludes the canonical Lin28a binding site at the 3' of the Luc-D2 construct; the mutant Luc-D2 Δ2 is deleted also of the 5' region of Luc-D2 containing a possible variant of the canonical Lin28a binding site (GGTGCT). Data on luciferase activity, normalized to Renilla luciferase, are shown as mean ±SEM of fold changes relative to values measured in Mock transfected cells. P value of biological replicates (n=4) was calculated using Student's t-test (two tailed). *P<0.05; ns: not significant. (c) Western blot analysis of rescue experiment. After 1 day of EpiLC transition, the cells were co-transfected with Lin28a-Flag expressing plasmid or empty vector

(Mock) and control or specific siRNA as indicated. Endo. Lin28a indicates the band of the endogenous protein.

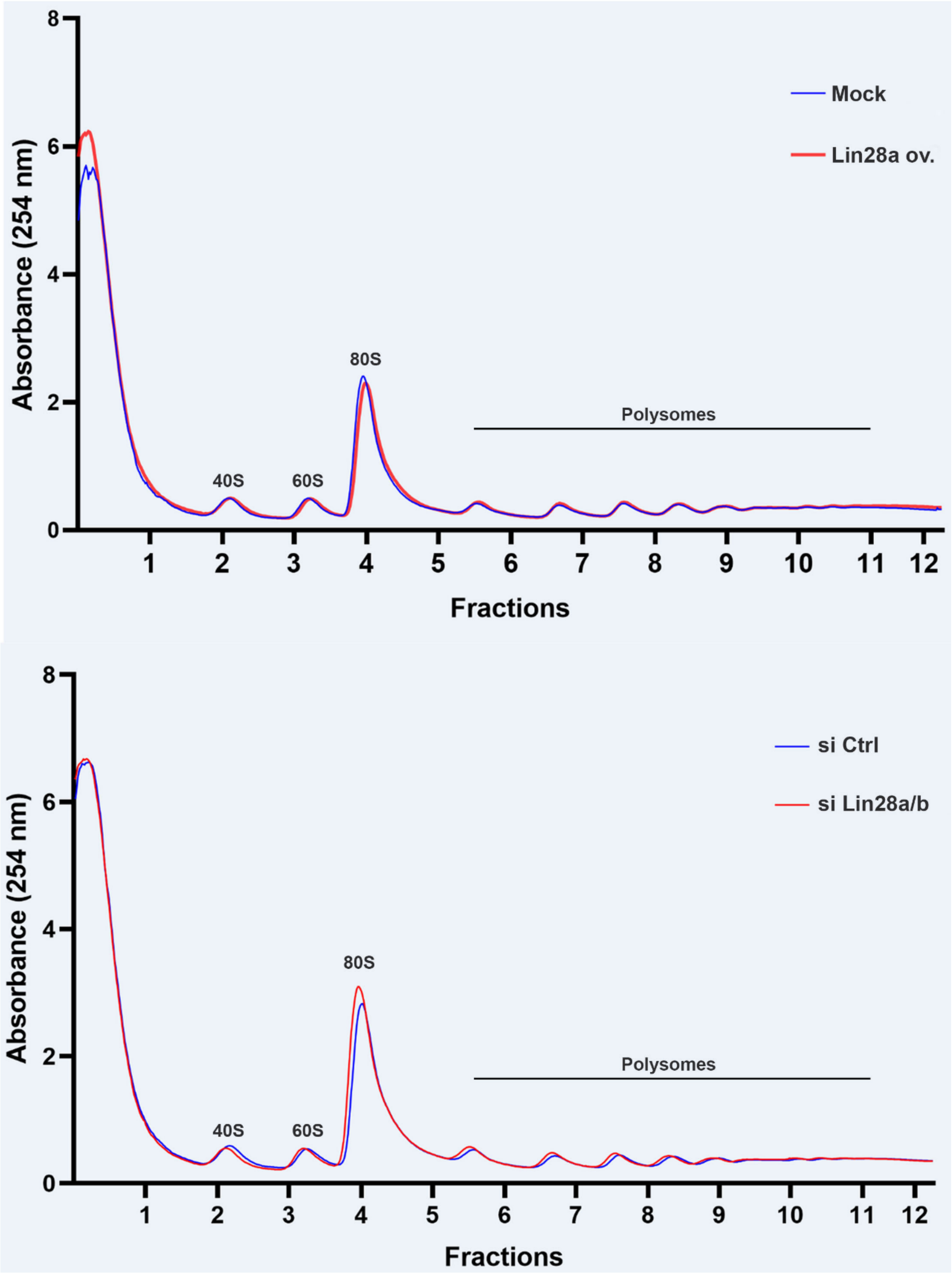

Supplementary Figure 3: Lin28a modulates Dnmt3a mRNA association to polysomes.

The graphs report polysome profiling upon overexpression or silencing of Lin28a. Polysome profiling absorbance indicates particle sedimentation in the different fractions. Ribosomal

subunits (60S and 40S) and monomers (80S) are eluted in fractions 3 to 5 whereas the polysomes in the fractions 6 to 12.

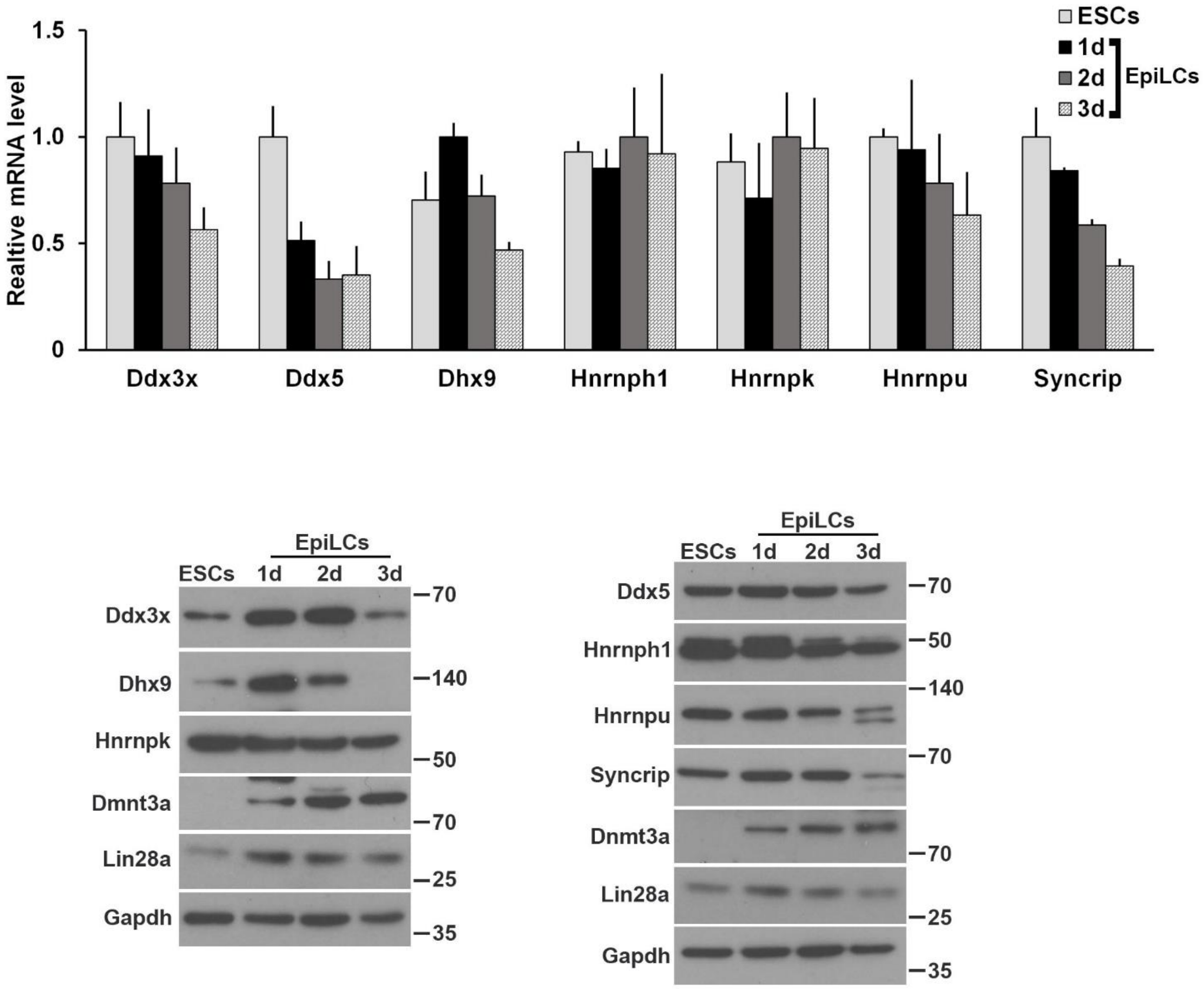

Supplementary Figure 4: Expression profile of Lin28a partners.
The expression profile of both mRNA and protein level of Lin28a partners was analyzed by qPCR and western blot at different time points of transition from ESCs to EpiLCs. The data in the graph are presented as fold changes relative to highest values ± SEM of three independent experiments.

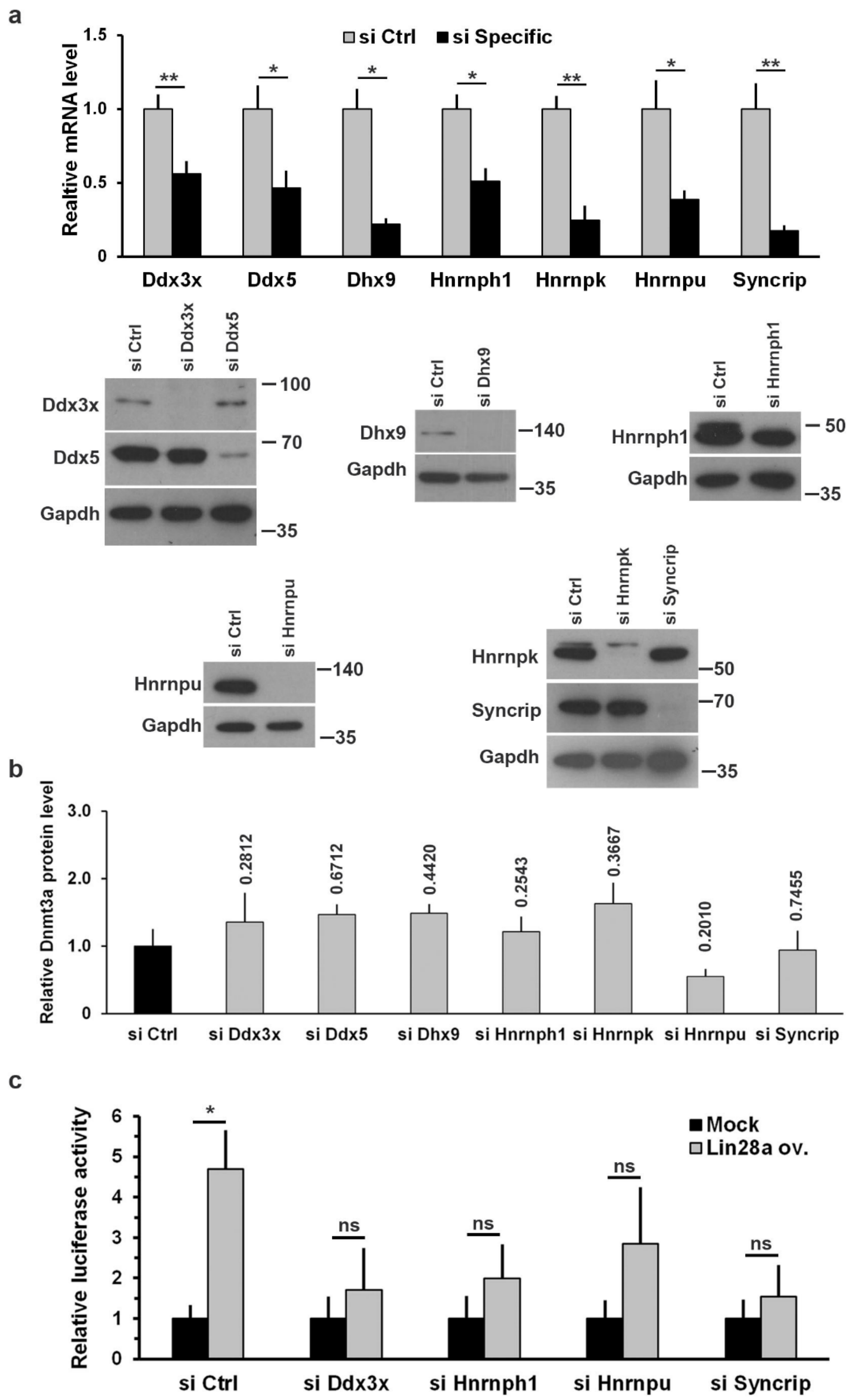

Supplementary Figure 5: Silencing of Lin28a partners.
(a) qPCR and western blot analysis to demonstrate the efficient silencing of Lin28a partners. The data in the graph are presented as mean ± SEM of the fold changes relative to values in control siRNA transfected cells of five independent experiments. *P<0.05; **P<0.005 (Student's t-test, two tailed). (b) Graph represents the changes of Dnmt3a protein level upon silencing of the indicated Lin28a partners. The relative protein levels are calculated as

Dnmt3a band intensity relative to Gapdh (n≥3 biological replicates). Data are shown as means±SEM of fold changes relative to values measured in control cells (n≥3). P values are reported on the relative bars (Student's t-test, two tailed). (c) Graph represents the luciferase activity using Luc-D2 construct upon overexpression of Lin28a and silencing of the indicated interactors. Data on luciferase activity, normalized to Renilla luciferase, are shown as mean ±SEM of fold changes relative to values measured in Mock transfected cells. P value of biological replicates (n=3) was calculated using Student's t-test (two tailed). *P<0.05; ns: not significant.

Supplementary Table 1: Protein identification in Pool1a and b and effect of RNase treatment.

Accession	# Peptides CTR_pool1b Mascot	# Peptides LIN28_pool1a Mascot	# Peptides LIN28_pool1b Mascot	Absent from pool1 of RNase-treated sample	Present in pool2 of RNase-treated sample (shifted)	Present in pool3 of RNase-treated sample (shifted)
A0A0N4SV	0	6	5	Absent	Present	Present
A0A0R4J17	0	0	1			
A0A0R4J1E	0	4	2	Absent	Present	
A0A1B0GS	1	0	3	Absent		
A0A1S6GW	0	0	1	Absent		
A0A1S6GW	0	0	1	Absent		
A0A1S6GW	0	6	3		Present	
A0A1W2P7	0	2	6	Absent		
A0A1Y7VK	1	5	3	Absent		
A1IGX7	0	0	6			
A2ARV4	0	16	10			
A2ATU9	0	0	2	Absent	Present	
B2M0S2	0	1	1			
B2RQC6	0	20	17	Absent		
B2RSV4	0	1	2	Absent		
B2RWX2	0	1	0		Present	
B9EIU1	0	19	17	Absent		
B9EKP5	0	66	65	Absent		
C5H0E8	0	1	1	Absent		
E9PVA8	0	13	5			
E9Q7L0	0	0	1			
E9Q8Z8	0	0	2			
E9Q9C3	0	1	1			
E9QAQ7	0	2	2			
E9QB02	0	7	9	Absent	Present	
E9QKR0	0	0	2	Absent		
E9QPX1	0	4	2			
G5E866	0	1	0			
G5E902	1	5	6	Absent		Present
O08528	0	0	1	Absent	Present	
O35593	0	2	1	Absent		
O55029	0	1	4			
O88477	0	4	2			
P07309	0	4	3	Absent		Present
P08752	1	1	2	Absent		
P11247	1	0	5	Absent		
P11440	0	0	2	Absent		Present
P12382	0	1	5		Present	
P14131	1	5	6	Absent		
P14685	0	1	2			
P16125	1	0	2		Present	
P17918	0	3	4			
P23116	0	1	2			
P26443	0	0	1	Absent	Present	
P30285	0	0	1	Absent	Present	Present
P38647	0	2	2			
P43247	0	2	1		Present	
P47857	0	0	2	Absent	Present	
P47963	0	2	1			
P48962	1	2	4	Absent	Present	
P53026	1	3	3	Absent		
P54071	0	0	2	Absent		Present
P54775	0	2	4	Absent		
P56546	0	2	4			
P60335	1	3	2	Absent		
P62245	1	3	6	Absent		
P62301	1	3	3	Absent		
P99027	1	3	2	Absent		
Q14AQ1	0	3	8			
Q3T9L0	0	0	1	Absent		
Q3T9Y8	0	2	2			
Q3TF14	1	0	5	Absent	Present	
Q3TF62	0	6	3	Absent		
Q3TF87	0	7	9	Absent		
Q3TFA9	0	0	1	Absent		
Q3TG52	0	0	1	Absent	Present	
Q3TGU7	0	0	1	Absent		Present
Q3THA0	0	0	2			
Q3THE2	1	2	0	Absent		
Q3THS6	0	0	1	Absent	Present	
Q3THW7	0	0	1			
Q3TI61	0	3	5			
Q3TIN2	0	8	7	Absent	Present	
Q3TIQ2	1	2	5	Absent		
Q3TJH1	1	2	2	Absent		
Q3TK29	0	0	2	Absent		
Q3TL71	0	0	2	Absent		
Q3TMA0	0	1	0			
Q3TQ70	0	0	2	Absent		
Q3TQX5	0	0	3	Absent		
Q3TVV6	0	9	3			
Q3TW74	0	1	4	Absent		Present
Q3U2W2	0	2	2			
Q3U3D3	0	1	4		Present	
Q3U6P5	0	2	2	Absent	Present	
Q3U741	0	1	1		Present	
Q3U8X1	0	3	6			
Q3UD06	0	6	6	Absent		
Q3UH59	0	6	1	Absent		
Q3UI84	0	2	3			
Q3UIG0	0	1	4			
Q3UIQ2	0	1	1			
Q3ULS2	0	4	4			
Q3UNH3	0	0	2	Absent		
Q3UWW9	0	3	7	Absent		
Q3UXP2	0	5	3			
Q3UXQ6	0	2	4	Absent		
Q3UZG4	0	2	5	Absent		
Q3V0Z8	0	3	1			
Q3V122	0	0	1		Present	
Q3V3R1	0	0	4	Absent	Present	
Q4FZE6	0	4	3	Absent		
Q4VAG4	0	2	2	Absent		
Q52KC3	0	0	1		Present	
Q5BLJ9	0	4	5	Absent		
Q5BLK2	0	4	2	Absent	Present	
Q5FWB6	0	5	4	Absent		
Q5M9P3	0	5	8	Absent		
Q61768	0	0	3			
Q61990	1	1	2			
Q62318	0	11	9	Absent	Present	
Q6GQT9	0	3	4			
Q6NZJ6	0	1	0			
Q6P5F9	0	1	1			
Q6ZPT2	0	8	10	Absent		
Q6ZQK2	0	4	3	Absent		
Q7TQI3	0	0	1	Absent		
Q80TT4	0	1	1		Present	
Q80UL3	0	1	1			
Q80X90	0	5	6	Absent		
Q80ZI7	0	1	1			
Q8BHC4	0	3	1			
Q8BKC5	0	4	7	Absent	Present	
Q8BKG3	0	7	4			
Q8BP47	0	0	1			
Q8BTF0	0	5	2			
Q8BU30	0	7	8	Absent		
Q8C338	1	0	3	Absent	Present	
Q8C605	0	0	2	Absent	Present	
Q8CG47	0	2	2			
Q8CIG8	0	0	1		Present	
Q8CIJ3	0	0	1			
Q8K2T1	0	1	1	Absent	Present	
Q8QZY1	0	2	5			
Q8R010	0	3	4	Absent		
Q8R1B4	0	1	0			
Q8VDD5	1	20	16	Absent		
Q8VHX6	0	5	5			
Q91V55	0	5	3	Absent		
Q91XE1	0	1	1			
Q921F2	0	2	2	Absent	Present	
Q99PV0	0	1	1			
Q9CQZ5	0	2	1			
Q9CXW2	0	1	0			
Q9D051	0	1	1	Absent	Present	Present
Q9D0I9	0	11	9	Absent		
Q9D1M4	0	1	0	Absent		
Q9D1N9	0	2	0			
Q9D7S7	0	0	1			
Q9D8N0	1	2	6	Absent		
Q9DB05	0	0	1	Absent		
Q9DB20	1	6	5	Absent		
Q9DB79	0	4	5	Absent		
Q9DBZ5	0	2	1	Absent		
Q9DCX2	1	4	4	Absent	Present	
Q9ERK4	0	0	2			
Q9JHU4	0	13	18			
Q9JIF0	0	0	1			Present
Q9JIF7	0	5	9	Absent		
Q9JJI8	1	3	3	Absent		
Q9JKF7	0	1	0			
Q9QXX4	0	2	2		Present	
Q9QYF1	0	1	2			
Q9WUK4	0	3	3			
Q9Z2I8	0	4	0			
Q9Z2X1	0	1	1	Absent	Present	
V9GX06	1	1	1	Absent		

Supplementary Table 2: Protein identification in Pool2 fraction and effect of RNase treatment.

Accession	Score CTR_pool2 Mascot	Score LIN28_pool2 Mascot	# Peptides CTR_pool2 Mascot	# Peptides LIN28_pool2 Mascot	Absent from pool2 of RNase-treated sample	Present in pool3 of RNase-treated sample (shifted)	Accession	Score CTR_pool2 Mascot	Score LIN28_pool2 Mascot	# Peptides CTR_pool2 Mascot	# Peptides LIN28_pool2 Mascot	Absent from pool2 of RNase-treated sample	Present in pool3 of RNase-treated sample (shifted)
A0A0R4J0W6		114	0	4			Q543C2		62	0	1		
A0A1S6GWH4	48	283	1	6			Q5SUR0		118	0	2		
B2RY26		45	0	1			Q5SW15		87	0	2		
C5H0E8	63	113	1	2	Absent		Q5SX39		45	0	1		
E9PYR1		97	0	1			Q61768	51	325	1	7	Absent	
E9Q8Z8	32	227	1	6	Absent		Q6NZJ6	50	61	1	3	Absent	
E9QKR0		123	0	2			Q6P5F9		221	0	1		
E9QNN1		48	0	1		Present	Q6ZQK2	43	423	1	11		
G3XA66	80	149	1	3			Q7TMK9		195	0	2		
G5E924	33	178	1	4	Absent		Q80X90		104	0	2	Absent	
O08553	53	100	1	3	Absent		Q8BFY9		593	0	8		
O35593		93	0	2			Q8BHC4		157	0	4	Absent	
O88477	89	599	1	7	Absent		Q8BJX0		33	0	1	Absent	
P62301		56	0	2	Absent		Q8BTF0		39	0	1	Absent	
Q3TF62		48	0	1	Absent		Q8C2Q7		33	0	1	Absent	
Q3TF87	29	40	1	3	Absent		Q8QZY1		170	0	3		
Q3THE2		180	0	2	Absent		Q8VDD5	90	176	1	7	Absent	
Q3THW7		36	0	1	Absent		Q8VHX6		97	0	1	Absent	
Q3TIX6		98	0	3	Absent		Q91V55	57	78	1	2	Absent	
Q3TMA0		72	0	3			Q99JX4		188	0	5	Absent	
Q3TQ70	27	98	1	2			Q99LC5		333	0	6		
Q3TQX5		71	0	2			Q99PV0		26	0	1	Absent	
Q3TVV6		34	0	1			Q9CQZ5		152	0	1		
Q3UH59		79	0	4	Absent		Q9CY16		33	0	1	Absent	
Q3UI84		39	0	1	Absent		Q9D2R0	67	80	1	3	Absent	
Q3UIQ2	89	119	1	2			Q9DB05	29	181	1	3		
Q3UK30		32	0	1			Q9ERK4	48	386	1	7		
Q3UXP2	61	131	1	5	Absent		Q9JIF7		101	0	2	Absent	
Q3V0Z8		209	0	3			Q9R0E1		70	0	1		
Q540E6		96	0	2	Absent	Present	Q9WUK4		239	0	4		
Q543C2		62	0	1									

Supplementary Table 3: Protein identification in Pool3 fraction and effect of RNase treatment.

Accession	Score CTR_pool3 Mascot	Score LIN28_pool3 Mascot	# Peptides CTR_pool3 Mascot	# Peptides LIN28_pool3 Mascot	pool3 of RNase-treated	Accession	Score CTR_pool3 Mascot	Score LIN28_pool3 Mascot	# Peptides CTR_pool3 Mascot	# Peptides LIN28_pool3 Mascot	from pool3 of RNase-
A0A0G2JGQ4		75	0	1	Absent	Q3UXP2		91	0	3	Absent
A0A0R4J0W6		112	0	4	Absent	Q3UZG3		73	0	1	
A0A1S6GWH		62	0	2	Absent	Q3V0Z8	61	368	1	5	Absent
B1AT82		151	0	3	Absent	Q5BLK2		41	0	1	Absent
B2RWX2		99	0	1		Q5SUR0		100	0	1	Absent
B2RY26		4214	0	54		Q5SX39		762	0	11	
C5H0E8		138	0	2		Q61768		84	0	3	Absent
D3YYI8		25	0	1	Absent	Q6P5F9		69	0	1	Absent
E9Q8Z8		87	0	2	Absent	Q6ZQK2		57	0	4	Absent
E9QKR0		43	0	1	Absent	Q7TMK9	36	347	1	4	Absent
G3XA66		40	0	1	Absent	Q8BFY9	83	381	1	6	Absent
G5E924		236	0	6	Absent	Q8BHC4		33	0	1	Absent
O35593	45	100	1	2	Absent	Q8BJX0		41	0	1	Absent
O88477		80	0	3	Absent	Q8BK37		79	0	3	Absent
P14131		42	0	1	Absent	Q8CGB9		272	0	8	
P26443		211	0	4	Absent	Q8VDD5		299	0	8	Absent
Q3TF87		28	0	1	Absent	Q8VEL2		32	0	1	Absent
Q3THA0		52	0	1	Absent	Q99LC5	30	222	1	3	Absent
Q3THE2		41	0	1	Absent	Q9CQZ5		100	0	1	Absent
Q3THW7		35	0	1	Absent	Q9D2Q8		326	0	1	Absent
Q3TIX6		173	0	5	Absent	Q9D2R0		82	0	1	Absent
Q3TQX5	31	164	1	5	Absent	Q9DB05		41	0	2	Absent
Q3UK30		371	0	5	Absent	Q9ERK4		57	0	2	Absent
Q3UK83		25	0	1	Absent	Q9JI91		52	0	2	
Q3UKW2		417	0	2		Q9R0E1		63	0	2	Absent

Supplementary Table 4: Proteins identified in more than one pool.

pools	total	Accession	pools	total	Accession	pools	total	Accession	pools	total	Accession
pool 1,2 and 3	19	Q6ZQK2	pool 1	126	Q8C338	pool 1 (continue)		A0A0N4SVP8	pool 2	9	Q99JX4
		Q3UXP2			Q3TFA9			Q9D051			Q8C2Q7
		Q8VDD5			P56546			Q921F2			Q5SW15
		O35593			Q3U2W2			E9QB02			Q9CY16
		Q9CQZ5			G5E866			A0A0R4J170			O08553
		Q61768			Q3V122			Q52KC3			E9PYR1
		E9Q8Z8			Q80UL3			Q9CXW2			Q540E6
		Q3V0Z8			A0A1Y7VKY1			Q3U3D3			Q543C2
		Q3THW7			Q61990			P62245			E9QNN1
		Q8BHC4			Q4VAG4			E9Q9C3	pool 3	11	Q8BK37
		Q9DB05			Q8R010			E9QPX1			B1AT82
		Q3TQX5			P23116			P16125			D3YYI8
		Q6P5F9			A0A0R4J1E2			B9EKP5			Q9D2Q8
		O88477			P43247			Q3U6P5			Q3UZG3
		Q3THE2			Q91XE1			Q3V3R1			Q9JI91
		E9QKR0			Q8BU30			Q3UWW9			Q8CGB9
		C5H0E8			Q5BLJ9			Q14AQ1			A0A0G2JGQ4
		Q3TF87			B2M0S2			Q8C605			Q3UKW2
		Q9ERK4			Q3UNH3			E9PVA8			Q8VEL2
pool 1 and 2	17	Q6NZJ6			A0A1B0GSX0			Q9D1M4			Q3UK83
		Q91V55			A2ARV4			P17918			
		Q99PV0			Q9JHU4			Q80ZI7			
		Q3TVV6			G5E902			Q9QYF1			
		Q9WUK4			Q3UD06			Q9DCX2			
		Q9JIF7			Q9QXX4			P48962			
		Q80X90			O55029			Q3UIG0			
		Q3UI84			P60335			O08528			
		Q3TQ70			Q8BP47			A1IGX7			
		P62301			B2RSV4			Q9DB79			
		Q8BTF0			P47963			Q3TL71			
		Q8QZY1			P08752			Q3TW74			
		Q3UH59			Q9DBZ5			Q9Z2X1			
		Q3UIQ2			Q3TGU7			Q8CIJ3			
		Q3TF62			A0A1S6GWJ8			P12382			
		Q8VHX6			P38647			A0A1S6GWH5			
		Q3TMA0			Q3ULS2			Q4FZE6			
pool 1 and 3	5	P26443			Q3TK29			Q3TF14			
		Q3THA0			Q8BKC5			Q9D7S7			
		Q5BLK2			Q3UZG4			P11247			
		B2RWX2			Q8R1B4			A0A1W2P7A1			
		P14131			P47857			Q3U8X1			
pool 2 and 3	15	Q3TIX6			Q9JJI8			Q6ZPT2			
		Q8BJX0			Q9D1N9			Q3T9L0			
		A0A1S6GWH4			Q9DB20			Q80TT4			
		Q9R0E1			P54775			Q9D8N0			
		Q3UK30			Q3TG52			A2ATU9			
		A0A0R4J0W6			Q9Z2I8			Q3TJH1			
		Q5SX39			A0A1S6GWH2			Q3T9Y8			
		B2RY26			Q9D0I9			Q3TI61			
		G5E924			Q62318			Q9JIF0			
		Q8BFY9			Q5M9P3			Q8CG47			
		Q99LC5			P14685			Q9JKF7			
		Q7TMK9			Q3U741			P30285			
		G3XA66			B2RQC6			V9GX06			
		Q9D2R0			P53026			Q3THS6			
		Q5SUR0			E9QAQ7			P99027			
					P11440			B9EIU1			
					Q8BKG3			P07309			
					Q8K2T1			Q3TIQ2			
					Q3TIN2			E9Q7L0			
					Q7TQI3			P54071			
					Q8CIG8			Q6GQT9			
					Q5FWB6			Q3UXQ6			

Supplementary Table 5: Overall identification of Lin28a co-purified proteins.

Accession	Gene Name	Accession	Gene Name	Accession	Gene Name	Accession	Gene Name
Q9D2R0	AACS	Q99LC5	ETFA	Q9D1N9	MRPL21	Q9WUK4	RFC2
Q3TF62	ACTL6A	E9PYR1	FBXL18	Q9JKF7	MRPL39	Q3UI84	RFC4
Q9JI91	ACTN2	B9EKP5	FLNA	Q9CXW2	MRPS22	Q540E6	RHEB
E9Q9C3	AFDN	Q80X90	FLNB	Q9CY16	MRPS28	P53026	RPL10A
Q3UZG4	AIMP1	Q8VHX6	FLNC	P43247	MSH2	Q3TIQ2	RPL12
Q8R010	AIMP2	Q3TIX6	FUBP3	Q3TW74	MTHFD1	P47963	RPL13
Q3TG52	ALDH9A1	Q80UL3	GALK1	Q3V3R1	MTHFD1L	Q4VAG4	RPL22
E9QAQ7	ARID1A	E9PVA8	Gcn1l1	Q8VEL2	MTMR14	Q9D7S7	RPL22L1
Q3UD06	ATP5C1	P26443	GLUD1	G3XA66	MTX1	Q5BLJ9	RPL27
Q9DCX2	ATP5H	D3YYI8	Gm10093	Q3U2W2	MYBBP1A	Q9JJI8	RPL38
Q9DB20	ATP5O	V9GX06	Gm11214	Q3UH59	MYH10	Q5FWB6	RPLP0
B2RWX2	C4B	A0A1Y7VKY1	GM11361	Q5SX39	MYH4	P99027	RPLP2
B2RQC6	CAD	Q3TF14	GM4737	B2RY26	MYH7	Q9DB79	RPS11
Q3UKW2	CALM1	A0A0N4SVP8	GM5580	Q8VDD5	MYH9	A0A1W2P7A1	RPS12
P11440	CDK1	C5H0E8	GM9392	Q3THE2	MYL12B	P62301	RPS13
P30285	CDK4	P08752	GNAI2	Q9DB05	NAPA	P62245	rps15a
B2M0S2	CLK2-SCAMP3	Q3TJH1	GNAI3	Q8BP47	NARS	P14131	RPS16
E9QPX1	COL18A1	Q3TQ70	GNB1	Q9CQZ5	NDUFA6	Q5M9P3	RPS19
Q8BTF0	COPA	E9QKR0	GNB2	Q3UIQ2	NDUFS1	Q5BLK2	RPS20
Q9JIF7	COPB1	Q3SX14	GSN	Q8K2T1	NMRAL1	Q3UXQ6	RPS4X
O55029	COPB2	A2ATU9	HAT1	Q6GQT9	NOMO1	Q91V55	RPS5
Q9ERK4	CSE1L	O08528	HK2	A0A1S6GWH4	NSUN2	Q4FZE6	RPS7
P56546	CTBP2	Q3UK83	HNRNPA1	A0A0G2JGQ4	NUB1	Q3UXP2	RUVBL2
E9Q8Z8	CTNND1	Q3UZG3	HNRNPA3	E9Q7L0	OGDHL	Q9D2Q8	S100A14
Q3TF87	DARS	Q3U6P5	HNRNPC	Q7TQI3	OTUB1	G5E866	SF3B1
Q8BHC4	DCAKD	Q9Z2X1	HNRNPF	Q3TGU7	PA2G4	B2RSV4	SF3B3
Q3U741	DDX17	Q8C2Q7	HNRNPH1	P60335	PCBP1	Q3TMA0	SLC16A3
Q3T9L0	DDX39	Q3TL71	HNRNPK	Q61990	PCBP2	Q9QXX4	SLC25A13
A0A1S6GWH2	DDX39B	G5E924	HNRNPL	P17918	PCNA	G5E902	SLC25A3
Q3TQX5	DDX3X	A0A1S6GWJ8	HNRNPM	Q9D051	PDHB	P48962	SLC25A4
Q3V0Z8	DDX5	Q3TVV6	HNRNPU	Q5SUR0	PFAS	A0A0R4J170	SMARCA4
E9QNN1	DHX9	P38647	HSPA9	P12382	PFKL	Q3ULS2	SMC2
O08553	DPYSL2	Q8BU30	IARS	P47857	PFKM	Q8CG47	SMC4
A1IGX7	DSG4	Q8CGB9	IDE	Q8C605	PFKP	Q9Z2I8	SUCLG2
Q9JHU4	DYNC1H1	Q8C338	IDH1	Q9R0E1	PLOD3	Q7TMK9	SYNCRIP
A0A0R4J1E2	EEF1D	P54071	IDH2	Q9JIF0	PRMT1	Q921F2	TARDBP
Q9D1M4	EEF1E1	O88477	IGF2BP1	Q8CIG8	PRMT5	Q3TFA9	TMOD3
Q9D8N0	EEF1G	Q8BKC5	IPO5	Q99PV0	PRPF8	Q8BFY9	TNPO1
P23116	EIF3A	Q6ZQK2	IQGAP1	B1AT82	PRPSAP1	Q80TT4	TOMM70a
Q8CIJ3	EIF3B	Q61768	KIF5B	Q8BK37	PRPSAP2	Q3TK29	TRAP1
Q8R1B4	EIF3C	Q6ZPT2	LARS	Q8BJX0	PSMB2	Q62318	TRIM28
Q3UIG0	EIF3E	A0A1B0GSX0	LDHA	P54775	PSMC4	P07309	TTR
Q3U8X1	EIF3F	P16125	LDHB	Q14AQ1	PSMC6	Q3UNH3	TXNL1
Q3THA0	EIF3G	A2ARV4	LRP2	Q3UWW9	PSMD11	A0A1S6GWH5	UBA1
Q3THW7	EIF3H	A0A0R4J0W6	LRRC40	O35593	PSMD14	Q543C2	UCK2
Q3T9Y8	EIF3I	E9QB02	MARS	Q3TI61	PSMD2	Q3UK30	Uncharacterized
Q9DBZ5	EIF3K	Q3THS6	MAT2A	P14685	PSMD3	Q3U3D3	VARS
Q8QZY1	EIF3L	Q52KC3	MCM5	Q8BKG3	PTK7	Q6P5F9	XPO1
Q99JX4	EIF3M	Q3V122	MCM7	Q3TIN2	QARS		
Q6NZJ6	EIF4G1	Q5SW15	METTL16	Q9D0I9	RARS		
B9EIU1	EPRS	P11247	MPO	Q9QYF1	RDH11		

Supplementary Table 6: Comparison of identified proteins with known arrays of RNPs.

HEK 293 RNPs (Trendel, 2019)			mESC RNPs (He, 2016)			miRNA BPs (Treiber, 2017)	P-bodies (Hubstenberger, 2017)	Stress granules (Jain, 2016)
AFDN	HNRNPU	RPL10A	ACTL6A	IGF2BP1	SYNCRIP	AIMP1	DHX9	CDK1
AIMP2	HSPA9	RPL12	ARID1A	IQGAP1	TARDBP	AIMP2	HNRNPC	CSE1L
CAD	IARS	RPL13	ATP5C1	MARS	TMOD3	DARS	HNRNPM	CTNND1
CDK4	IGF2BP1	RPL22	CALM1	MCM5	TNPO1	DDX3X	HNRNPU	DDX3X
COPA	IPO5	RPL22L1	COPA	MCM7	TOMM70a	EPRS	IGF2BP1	DPYSL2
COPB1	KIF5B	RPL27	CTBP2	MRPL21	TRAP1	HNRNPA1	MCM7	DYNC1H1
COPB2	LARS	RPL38	CTNND1	MRPL39	TRIM28	HNRNPA3	MYH10	EIF3A
CSE1L	LDHA	RPLP0	DARS	MRPS22	VARS	HNRNPF	RPS16	EIF3B
CTNND1	LDHB	RPLP2	DCAKD	MSH2		HNRNPH1	RPS4X	EIF3E
DARS	LRRC40	RPS11	DDX17	MTHFD1		HNRNPK	SF3B1	EIF3F
DDX17	MARS	RPS12	DDX39B	MTHFD1L		HNRNPL	SYNCRIP	EIF3G
DDX39B	MCM5	RPS13	DDX3X	MYBBP1A		HNRNPM		EIF3H
DDX3X	MCM7	RPS16	DDX5	MYH10		IARS		EIF3I
DDX5	METTL16	RPS19	DHX9	MYH9		IGF2BP1		EIF3K
DHX9	MRPL21	RPS20	DYNC1H1	MYL12B		LARS		EIF3L
DYNC1H1	MRPL39	RPS4X	EEF1D	NAPA		MARS		EIF3M
EEF1D	MRPS28	RPS5	EEF1E1	NDUFA6		PRMT1		EIF4G1
EEF1G	MSH2	RPS7	EEF1G	NDUFS1		QARS		FLNB
EIF3A	MTHFD1	RUVBL2	EIF3B	NSUN2		RARS		FUBP3
EIF3B	MTHFD1L	SF3B1	EIF3C	PA2G4		SF3B1		GNB2
EIF3C	MYBBP1A	SF3B3	EIF3E	PCNA		SF3B3		HNRNPA1
EIF3E	MYH10	SLC25A3	EIF3G	PLOD3		SYNCRIP		HNRNPA3
EIF3G	MYH9	SMARCA4	EIF3I	PRPF8				HNRNPK
EIF3H	NARS	SMC2	FLNA	PTK7				HSPA9
EIF3I	NDUFS1	SMC4	FLNB	RARS				IGF2BP1
EIF3L	NSUN2	SYNCRIP	FUBP3	RFC2				MARS
EIF3M	OTUB1	TARDBP	GCN1L1	RPL10A				MCM5
EIF4G1	PA2G4	TNPO1	GNAI2	RPS12				MCM7
EPRS	PCBP1	TRAP1	GNB1	RPS13				MTHFD1
ETFA	PCBP2	TRIM28	GNB2	RPL38				NSUN2
FLNA	PCNA	TXNL1	HK2	RPS11				PCBP2
FLNB	PFKL	UBA1	HNRNPA1	RPS12				PCNA
FUBP3	PFKM	VARS	HNRNPA3	RPS20				PRMT1
HK2	PRMT1	XPO1	HNRNPC	RPS5				PRMT5
HNRNPA1	PRMT5		HNRNPF	RUVBL2				PSMD2
HNRNPA3	PRPF8		HNRNPH1	SF3B1				RRC4
HNRNPC	PSMB2		HNRNPK	SF3B3				SMC4
HNRNPF	PSMD2		HNRNPL	SLC25A3				SYNCRIP
HNRNPH1	PSMD3		HNRNPM	SLC25A4				TARDBP
HNRNPK	PTK7		HNRNPU	SMARCA4				TMOD3
HNRNPL	QARS		HSPA9	SMC2				TNPO1
HNRNPM	RARS		IDH2	SMC4				UBA1

Supplementary Table 7: Proteins silenced by shRNAs in the Luc assay.

Gene name					
Aars	Ddx17	Gnai2	Myh10	Rfc4	Tnpo1
Actl6a	Ddx39a	Hat1	Myh4	Rheb	Tomm70a
Adk	Ddx39b	Hk2	Ndufs1	Rpl12	Trap1
Aimp2	Ddx3x	Hnrnph1	Nomo1	Rpl22l1	Trim28
Aldh9a1	Ddx5	Hnrnpk	Nsun2	Rpl27	Ttr
Arid1a	Dhx9	Hnrnpu	Pa2g4	Rpl38	Tufm
Atp1a1	Dync1h1	Igf2bp1	Pdhb	Rplp0	Txnl1
Atp2a2	Eef1e1	Impa1	Pfas	Rplp2	Uba1
Atp50	Eef1g	Ipo5	Pfkl	Rps11	Uck2
Atp5c1	Eif3a	Ldhb	Pfkm	Rps13	
Cad	Eif3b	Ltf	Pfkp	Rps16	
Cdk1	Eif3c	Map2k2	Phb	Rps20	
Cdk4	Eif3f	Mat2a	Pof1b	Rps4x	
Copa	Eif3h	Mat2b	Prmt1	Rps7	
Copb1	Eif3k	Mob2	Prmt5	Sae1	
Copb2	Eif3l	Mrpl21	Prpf8	Sf3b1	
Cse1l	Eif3m	Mrps22	Psmc6	Slc25a3	
Ctbp2	Eif4g1	Mrps28	Psmd11	Smarca4	
Ctnnd1	Etfa	Mthfd1	Psmd14	Smc4	
Dars	Glud1	Mybbp1a	Pzp	Syncrip	

Supplementary Table 8: Proteins whose silencing significantly affected Luc accumulation.

Gene name	Description
Aimp2	aminoacyl tRNA synthetase complex-interacting multifunctional protein 2(Aimp2)
Atp1a1	ATPase, Na+/K+ transporting, alpha 1 polypeptide(Atp1a1)
Atp5c1	ATP synthase, H+ transporting, mitochondrial F1 complex, gamma polypeptide 1(Atp5c1)
Cad	carbamoyl-phosphate synthetase 2, aspartate transcarbamylase, and dihydroorotase(Cad)
Copb2	coatomer protein complex, subunit beta 2 (beta prime)(Copb2)
Ddx3x	DEAD/H (Asp-Glu-Ala-Asp/His) box polypeptide 3, X-linked(Ddx3x)
Ddx5	DEAD (Asp-Glu-Ala-Asp) box polypeptide 5(Ddx5)
Dhx9	DEAH (Asp-Glu-Ala-His) box polypeptide 9(Dhx9)
Eif3k	eukaryotic translation initiation factor 3, subunit K(Eif3k)
Hnrnph1	heterogeneous nuclear ribonucleoprotein H1(Hnrnph1)
Hnrnpk	heterogeneous nuclear ribonucleoprotein K(Hnrnpk)
Hnrnpu	heterogeneous nuclear ribonucleoprotein U(Hnrnpu)
Igf2bp1	insulin-like growth factor 2 mRNA binding protein 1(Igf2bp1)
Map2k2	mitogen-activated protein kinase kinase 2(Map2k2)
Rplp0	ribosomal protein, large, P0(Rplp0)
Rps20	ribosomal protein S20(Rps20)
Syncrip	synaptotagmin binding, cytoplasmic RNA interacting protein(Syncrip)
Tomm70a	translocase of outer mitochondrial membrane 70 homolog A (yeast)(Tomm70a)
Trap1	TNF receptor-associated protein 1(Trap1)

Supplementary Table 9. List of primers used.

Name*	Application	Forward primer	Reverse primer
D1	PCR/Cloning	GATCGGTACCGGGACATGGGGGCAAACTGAAGTAGTG	CTAGGGTACCATTATGAAGTCGGGATGTACAGTAG
D2	PCR/Cloning	GATCGGTACCGATTTCACGTGTTTTAGCTGAGACATCG	CTAGGGTACCGCACAAAAACAGTCCATTTATTCAAGTG
D2 Δ1	PCR/Cloning	GATCGGTACCGATTTCACGTGTTTTAGCTGAGACATCG	CTAGGGTACCGTCACAAGCATCACGTACAC
D2 Δ2	PCR/Cloning	CTAGGGTACCCAGGACCTTCCAGAACCTTC	CTAGGGTACCGTCACAAGCATCACGTACAC
Lin28a ΔC	PCR/Cloning	GGTGGATCCGCCGCCATGGGCTCGGTGTCCAACCAGCAG	AGTCAAGCTTGCCATTCTGCGGGCCCTGCTGGGCCTTCAGTGG
Ddx3x	RT-qPCR	GCTGGCCTAGACCTGAACTC	ATAACGCCCTTTGCTTGCTG
Ddx5	RT-qPCR	GTGTAGCGAGACGAGAGACG	GGCCGGAGTCGCTTCAC
Dhx9	RT-qPCR	CCGACCACAACAGGAGCTTT	CGTGCGAAAATCCTTCTGCC
Dnmt3a	RT-qPCR<br>RIP-qPCR	CACCCCTGAGCCAGTAGGAG	GGCCATCCTCATACTCAGGC
Hnrnph1	RT-qPCR	AGTCCGTTAGGCAAAGTGGG	CTTTTCATCCGCGCTCCTGC
Hnrnpk	RT-qPCR	CGGAATCTGCCTCTTCCTCC	CCATGCCATCATAGCGGTCT
Hnrnpu	RT-qPCR	TACAGCAGAGCCAAGTCTCC	TGTGTCATCGAAGTGTTCGTCT
miR-23a	RIP-qPCR	ACTGGTGCATTCGGAAACCT	GGAGCATTCTTGCTTGCCTG
Syncrip	RT-qPCR	TGGAGGACCACCTCCAGATT	TCCCCACAAATATCTCAGTGCC
Fgf5	RT-qPCR	TCCATGCAAGTGCCAAATTTACGGA	TTCTGTGGATCGCGGACGCA
Gapdh	RT-qPCR	GTATGACTCCACTCACGGCAAA	TTCCCATTCTCGGCCTTG
Lin28a	RT-qPCR	GTTCGGCTTCCTGTCTATGACC	CTTCCATGTGCAGCTTGCTCT
Lin28b	RT-qPCR	GAGATAGGTGGAGACGGCAG	TTCTTCTCGCACAGTCCACA
Nanog	RT-qPCR	TCAGAAGGGCTCAGCACCA	GCGTTCACCAGATAGCCCTG
Rex1	RT-qPCR	GCAGTTTCTTCTTGGGATTTCAG	CTAATGCCCACAGCGAT

*The primer name is reported as indicated in the Figures.

RIP: RNA immunoprecipitation

Full-length blot relative to the Figures.

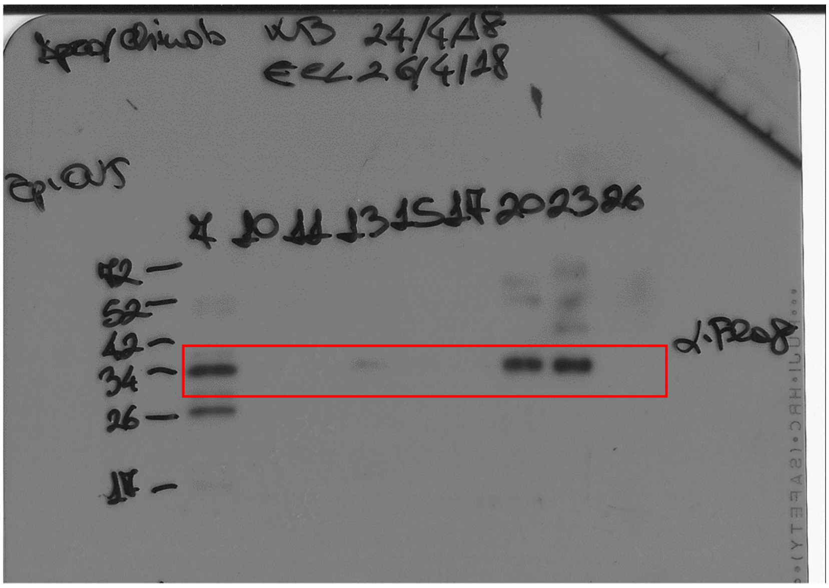

Full-length blot relative to Figure 1a. Red box indicates which part of the blot is assembled in the main figure.

A

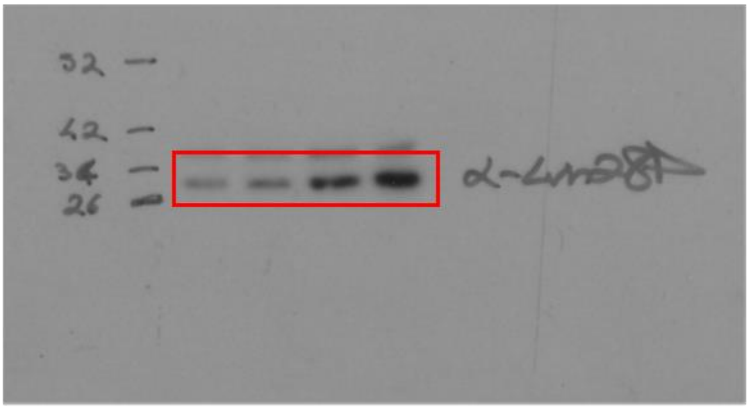

B

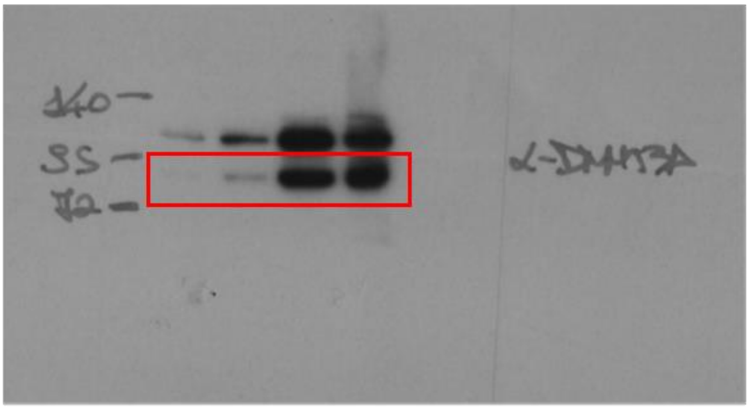

C

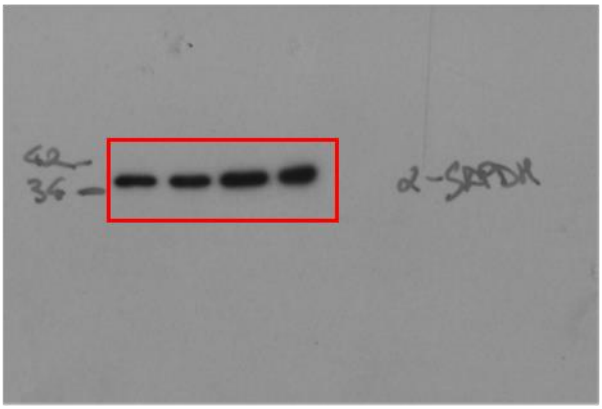

Full-length blots relative to Figure 2a. The blot in A is relative to Lin28a; the blot in B is relative to Dnmt3a; the blot in C is relative to Gapdh. Red boxes indicate which part of the blot is presented in the main figure.

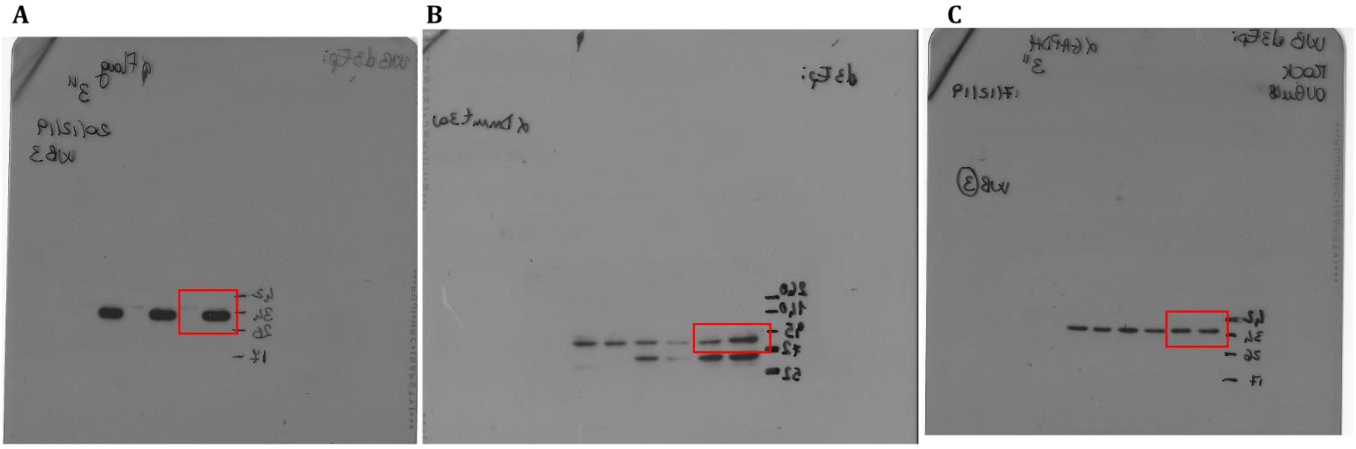

Full-length blots relative to Figure 2f. The blot in A is relative to Flag-tag of Lin28a-Flag; the blot in B is relative to Dnmt3a; the blot in C is relative to Gapdh. Red boxes indicate which part of the blot is presented in the main figure.

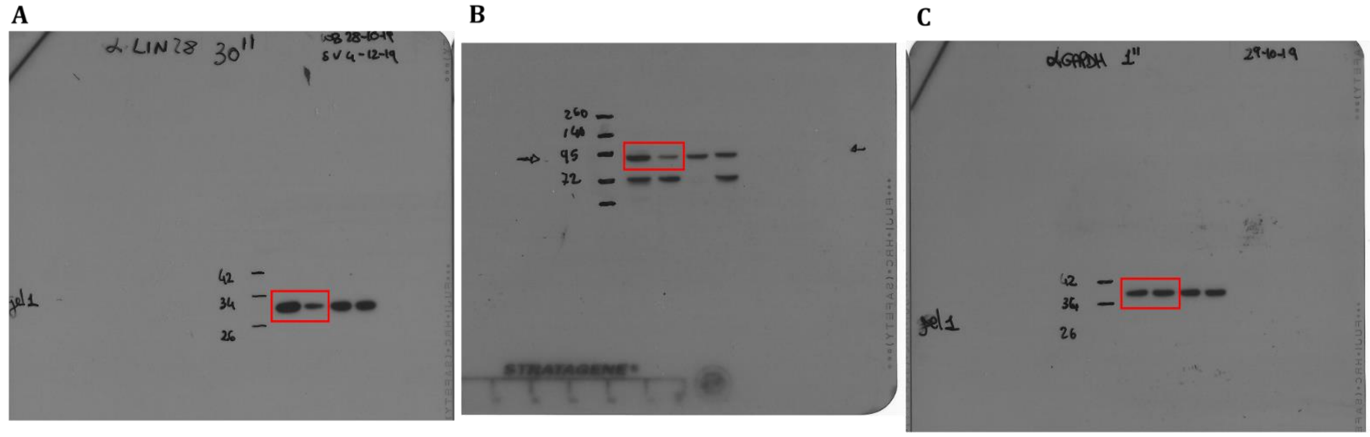

Full-length blots relative to Figure 2g. The blot in A is relative to Lin28a; the blot in B is relative to Dnmt3a; the blot in C is relative to Gapdh. Red boxes indicate which part of the blot is presented in the main figure.

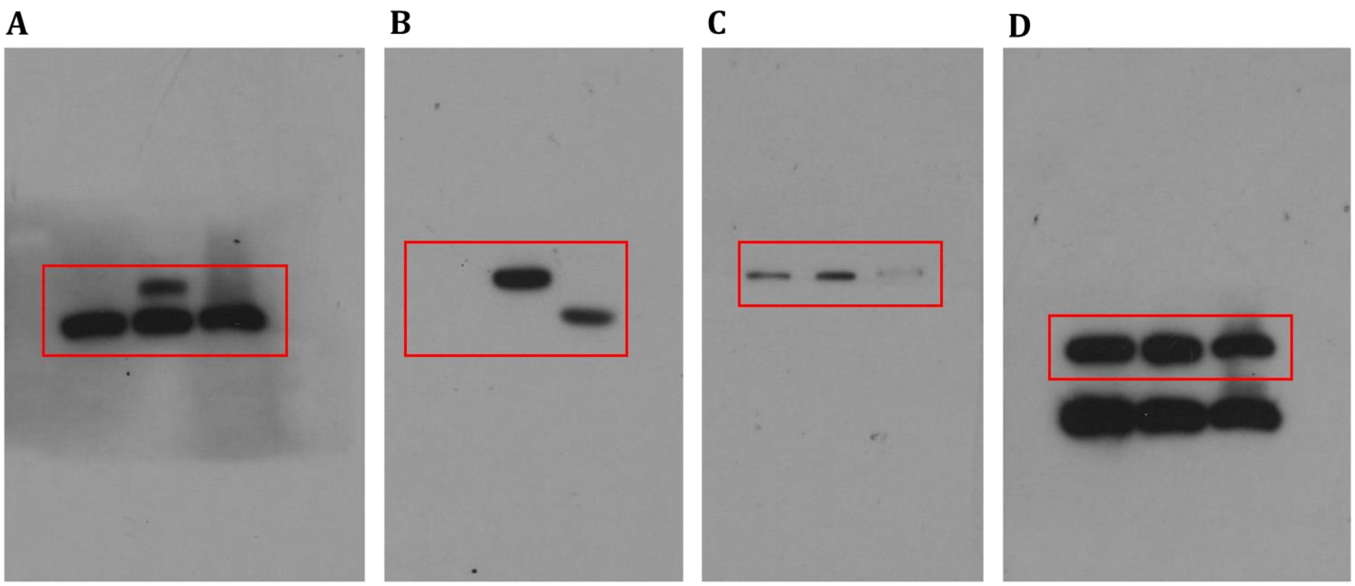

Full-length blots relative to Figure 3c. The blot in A is relative to Lin28a; the blot in B is relative to the Flag-tagged proteins; the blot in C is relative to Dnmt3a; the blot in D is relative to Gapdh. Red boxes indicate which part of the blot is presented in the main figure.

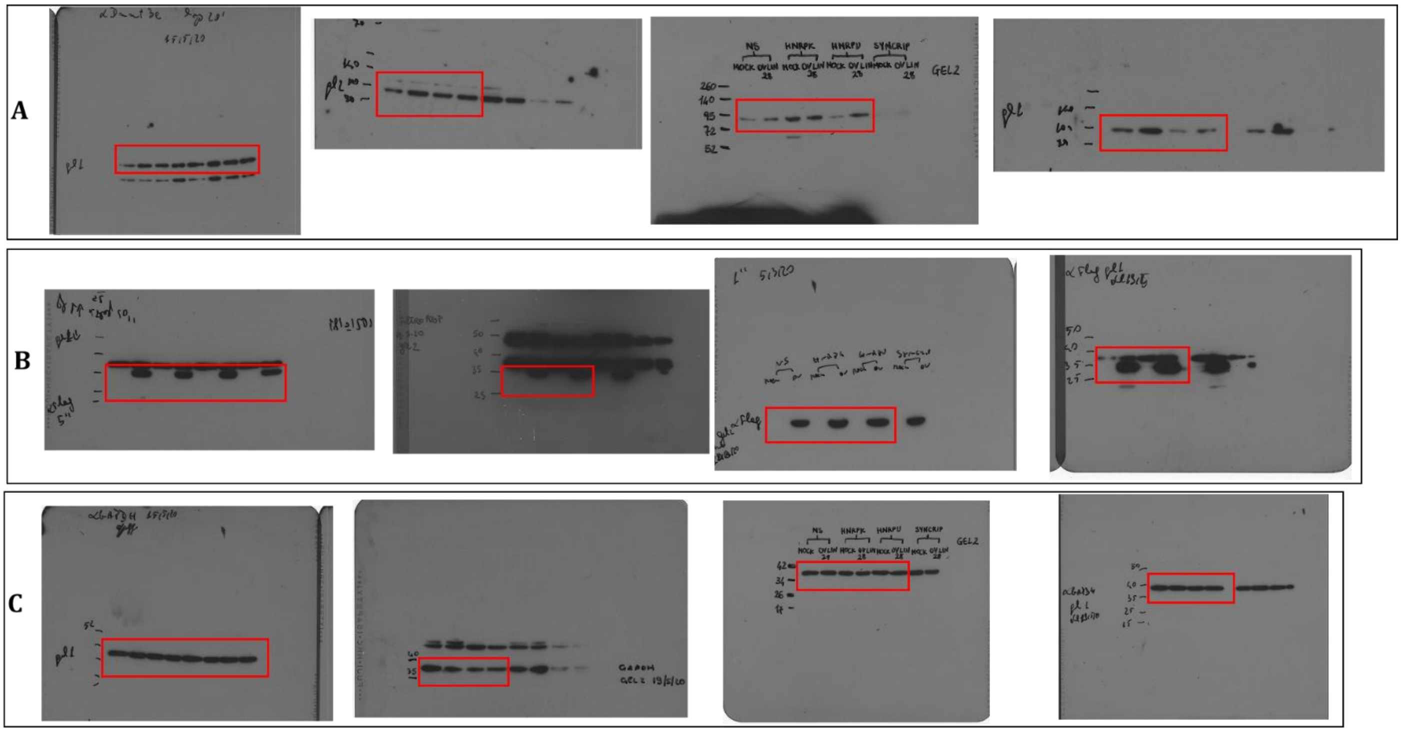

Full-length blots relative to Figure 5a. The line A is relative to Dnmt3a for the four series of blots assembled in the main figure; the line B is relative to Flag-tag for Lin28a-Flag; the line C is relative to Gapdh. Red boxes indicate which part of the blot is presented in the main figure.

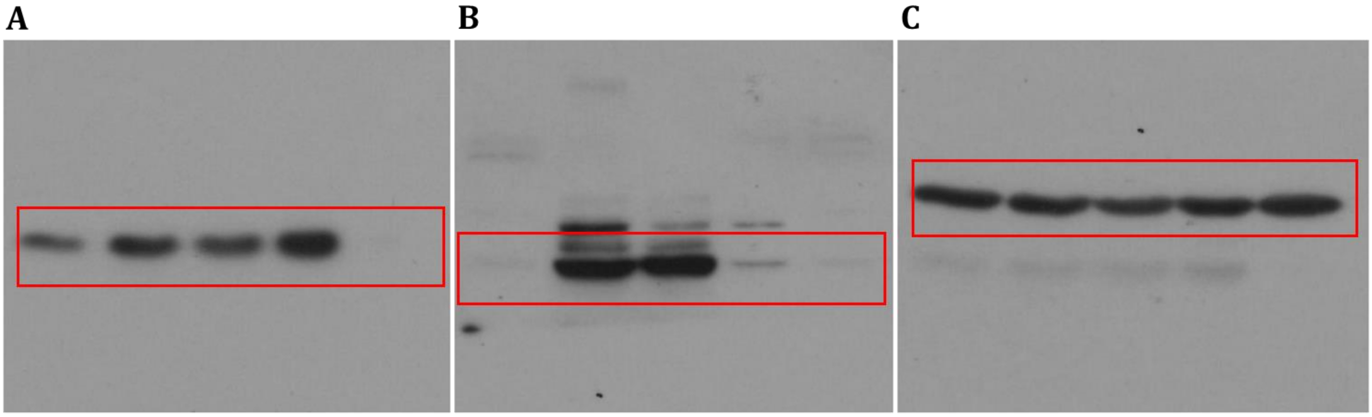

Full-length blots relative to Supplementary Figure 1a. The blot in A is relative to Lin28a; the blot in B is relative to Dnmt3a; the blot in C is relative to Gapdh. Red boxes indicate which part of the blot is presented in the main figure.

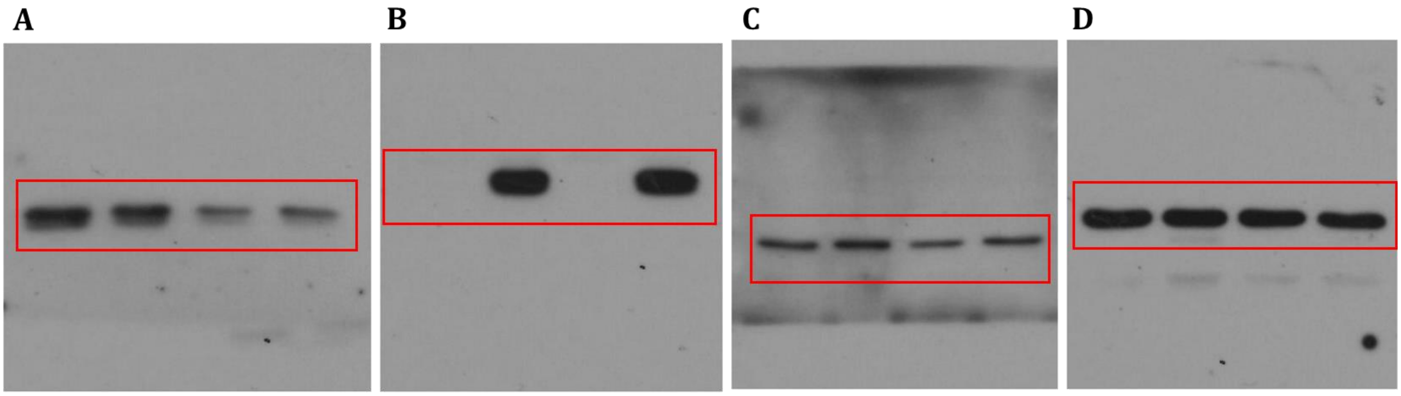

Full-length blots relative to Supplementary Figure 1c. The blot in A is relative to Lin28a; the blot in B is relative to Flag-tag of Lin28a-Flag; the blot in C is relative to Dnmt3a; the blot in D is relative to Gapdh. Red boxes indicate which part of the blot is presented in the main figure.

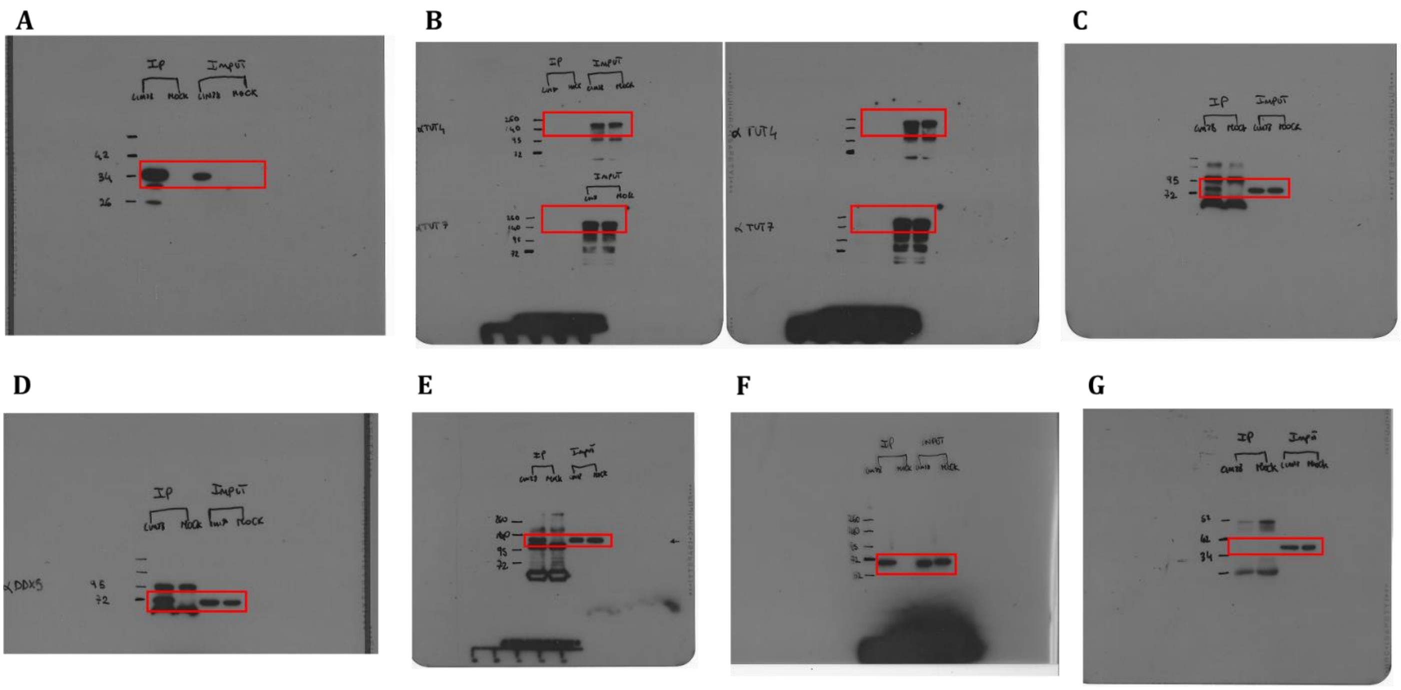

Full-length blots relative to Supplementary Figure 3. The blot in A is relative to the Flag-tag of Lin28a-Flag; the blot in B is relative to Tut4 and Tut7 (as indicated) for the two different exposures reported in the main figure; the blot in C is relative to Ddx3; the blot in D is relative to Ddx5; the blot in E is relative to Hnrnpu; the blot in F is relative to Syncrip; the blot in G is relative to Gapdh. Red boxes indicate which part of the blot is presented in the main figure.

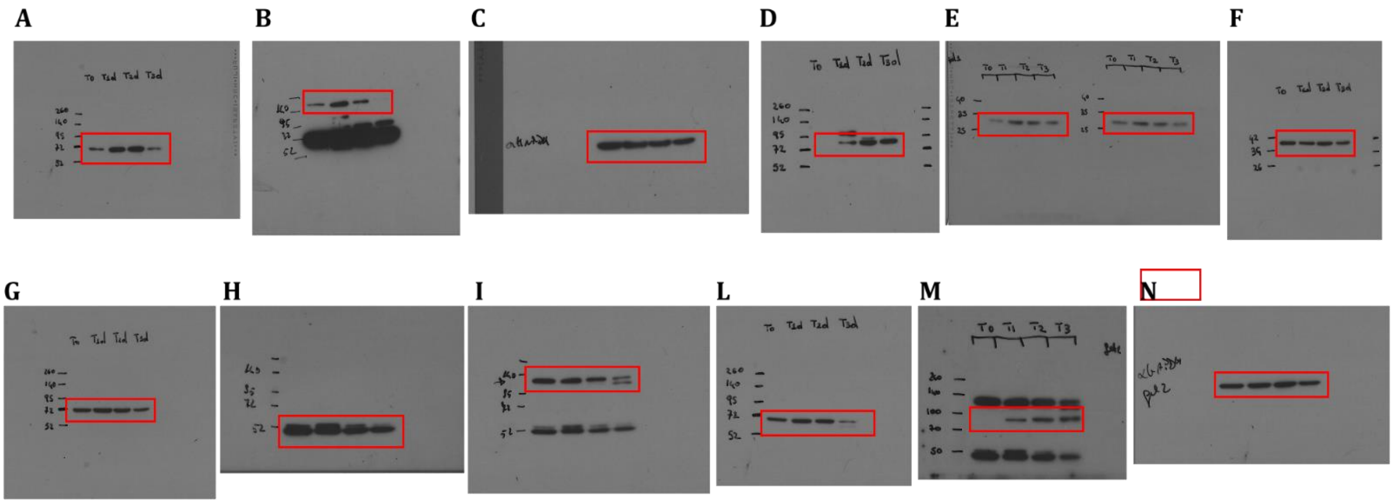

Full-length blots relative to Supplementary Figure 4. The blot in A is relative to Ddx3 (left gels); the blot in B is relative to Dhx9 (left gels); the blot in C is relative to Hnrnpk (left gels); the blot in D is relative to Dnmt3a (left gels); the blot in E is relative to Lin28a (for both left and right series of gels); the blot in F is relative to Gapdh (right gels); the blot in G is relative to Ddx5 (right gels); the blot in H is relative to Hnrnph1 (right gels); the blot in I is relative to Hnrnpu (right gels); the blot in L is relative to Syncrip (right gels); the blot in M is relative to Dnmt3a (right gels); the blot in N is relative to Gapdh (right gels). Red boxes indicate which part of the blot is presented in the main figure.

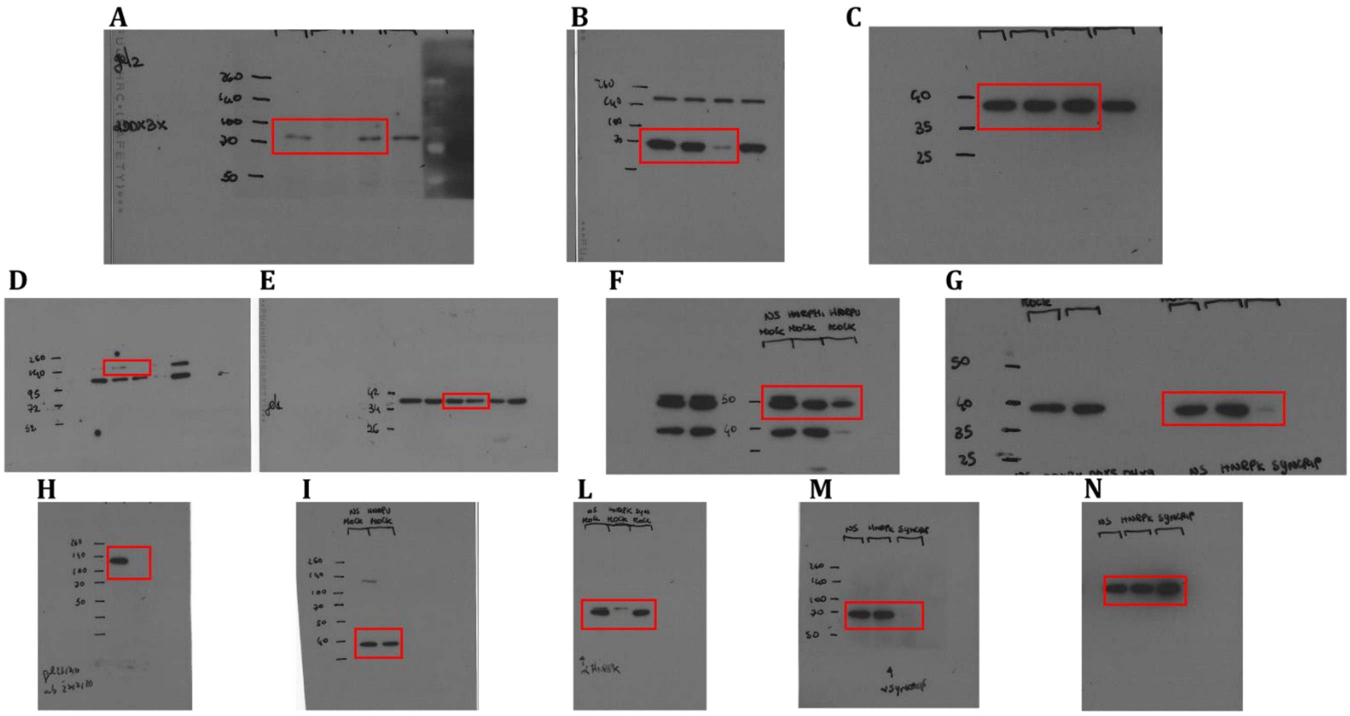

Full-length blots relative to Supplementary Figure 5a. The blots in A, B and C are relative to Ddx3, Ddx5 and Gapdh respectively; the blots in D and E are relative to Dhx9 and Gapdh respectively; the blots in F and G are relative to Hnrnph1 and Gapdh respectively; the blots in H and I are relative to Hnrnpu and Gapdh respectively; the blots in L, M and N are relative to Hnrnpk, Syncrip and Gapdh respectively. Red boxes indicate which part of the blot is presented in the main figure.
